# Supplementary material for: Evolutionary characterization of lung cancer metastasis
Source: Nature. 2026 Apr 29;653(8115):911–22. doi: 10.1038/s41586-026-10428-4 (PMC13190308; doi:10.1038/s41586-026-10428-4)
Supplement: Supplementary file 1 — This file contains Supplementary Figs. 1–10, Supplementary Table 1 and Supplementary Note. [file 41586_2026_10428_MOESM1_ESM.pdf]

nature portfolio

https://doi.org/10.1038/s41586-026-10428-4

Supplementary information

Evolutionary characterization of lung cancer metastasis

In the format provided by the
authors and unedited

Supplementary Information

Contents

Supplementary Figures 2

Supplementary Figure 1. Flow chart of patient recruitment to the TRACERx and PEACE studies. 2

Supplementary Figure 2. Anatomical and phylogenetic relationships of subclones. 3

Supplementary Figure 3. Comparison of the TRACERx-PEACE and TRACERx 421 Relapse cohorts. 4

Supplementary Figure 4. Relationship between systemic treatment and primary-metastasis genetic divergence. 5

Supplementary Figure 5. Site-specific mutational signature activity. 6

Supplementary Figure 6. The origin and location of migrations involving single or multiple tumour subclones. 7

Supplementary Figure 7. Comparison of metastatic seeding patterns inferred using different algorithms. 8

Supplementary Figure 8. SNVs and SCNA acquisition rate in seeding and non-seeding subclones. 9

Supplementary Figure 9. Driver alterations and seeding. 10

Supplementary Figure 10. SNV burden of seeding and non-seeding subclones in the TRACERx 421 relapse cohort stratified by relapse site. 11

Supplementary Tables 12

Supplementary Table 1. Clinical characteristics of patients in the TRACERx-PEACE Lung cohort 12

Supplementary Notes 13

1 Anatomical classifications informed by radiological imaging review 13

Supplementary Figures

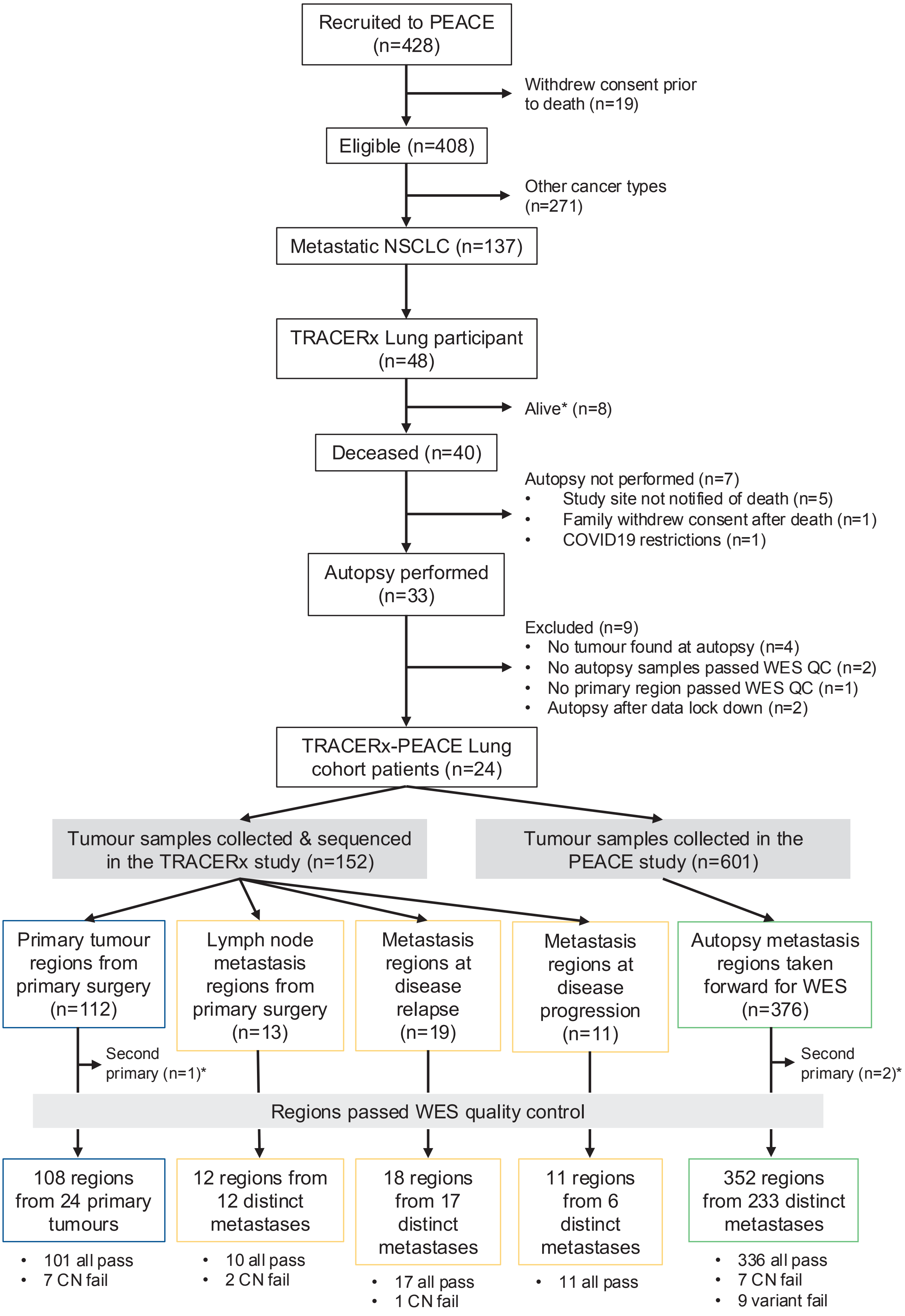

Supplementary Figure 1. Flow chart of patient recruitment to the TRACERx and PEACE studies. The number of primary (blue), pre-mortem metastasis (yellow) and post-mortem metastasis (green) samples collected, subjected to whole exome sequencing (WES), and that passed quality control (QC) are shown for the 24 patients that comprise the TRACERx-PEACE Lung cohort. NSCLC: non-small cell lung cancer.

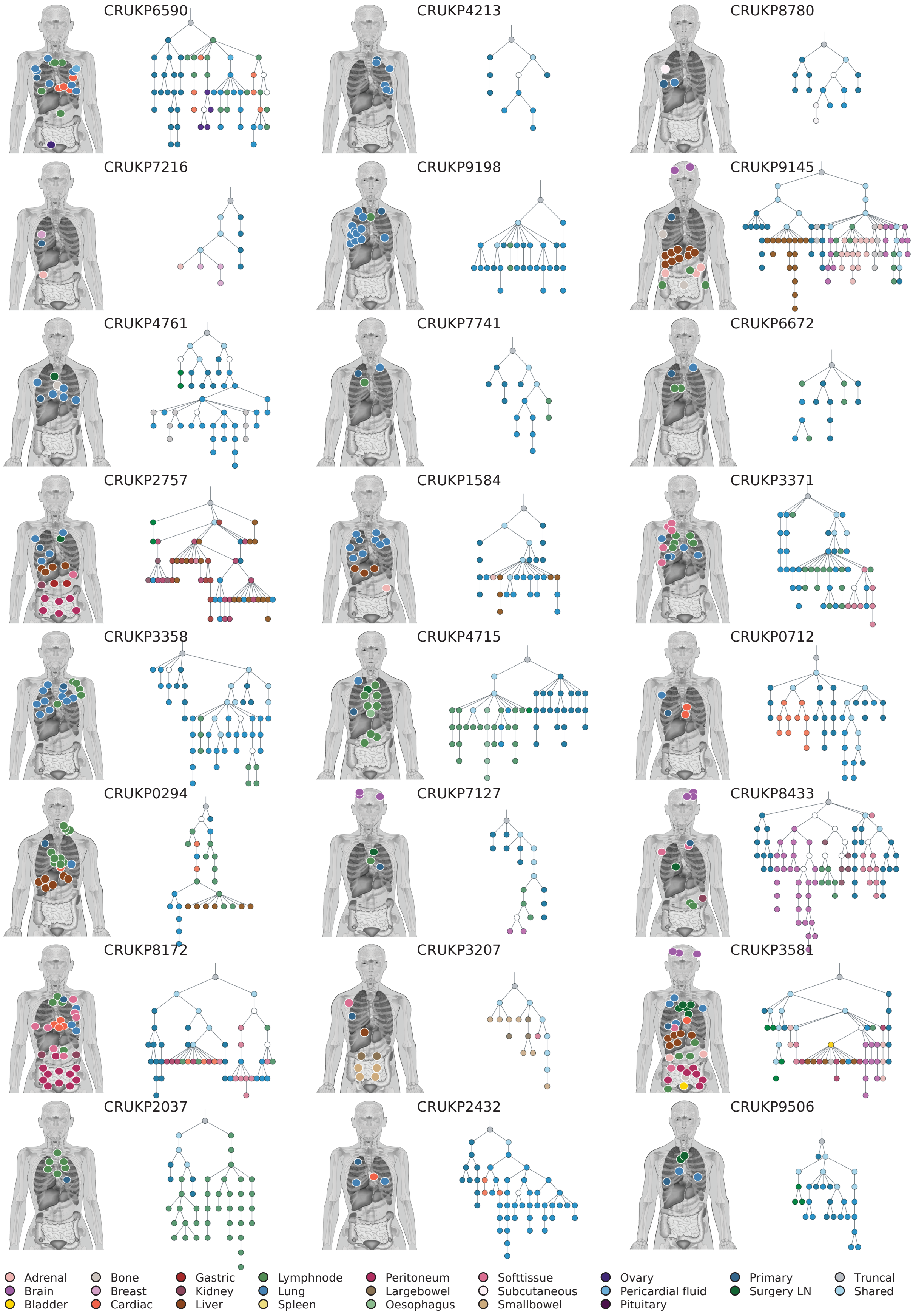

Supplementary Figure 2. Anatomical and phylogenetic relationships of subclones. The body maps depict the anatomical location of the primary and metastasis samples for each patient. Subclones (nodes) found only in metastasis samples (metastasis-unique) are coloured according to the anatomical location of the metastasis they were most prevalent in. Subclones found within the primary tumour are coloured based on their truncal (grey), shared subclonal (lightest blue) or primary-unique (darkest blue) classification (Methods). Subclones not prevalent in a specific sample are white.

a

	Tx-PEACE	Tx421-Relapse
n Patients	24	126
	**Mean regions per patient (range)**	
Primary	4.5 (2-8)	3.8 (1-8)
Metastasis	16 (3-39)	1.7 (1-6)

b

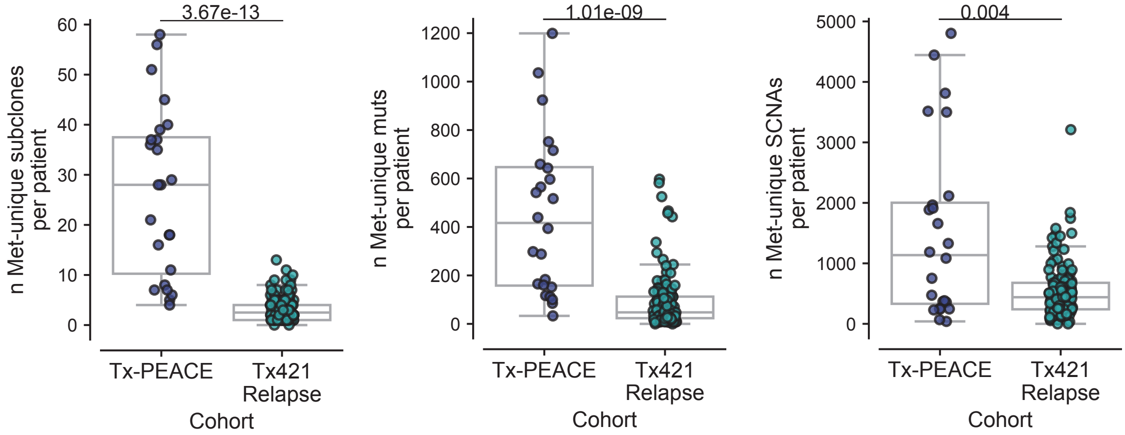

Supplementary Figure 3. Comparison of the TRACERx-PEACE and TRACERx 421 Relapse cohorts. a, Overview of the number of primary and metastasis regions available per patient in each cohort, **b,** Comparison of the number of metastasis-unique subclones, somatic mutations (muts) and copy number alterations (SCNAs) analyzed per patient in the TRACERx(Tx)-PEACE (purple) and TRACERx 421 Relapse (teal) cohorts. The box plots show the median and IQR with whiskers denoting values within 1.5 times the IQR from the first and third quartiles.

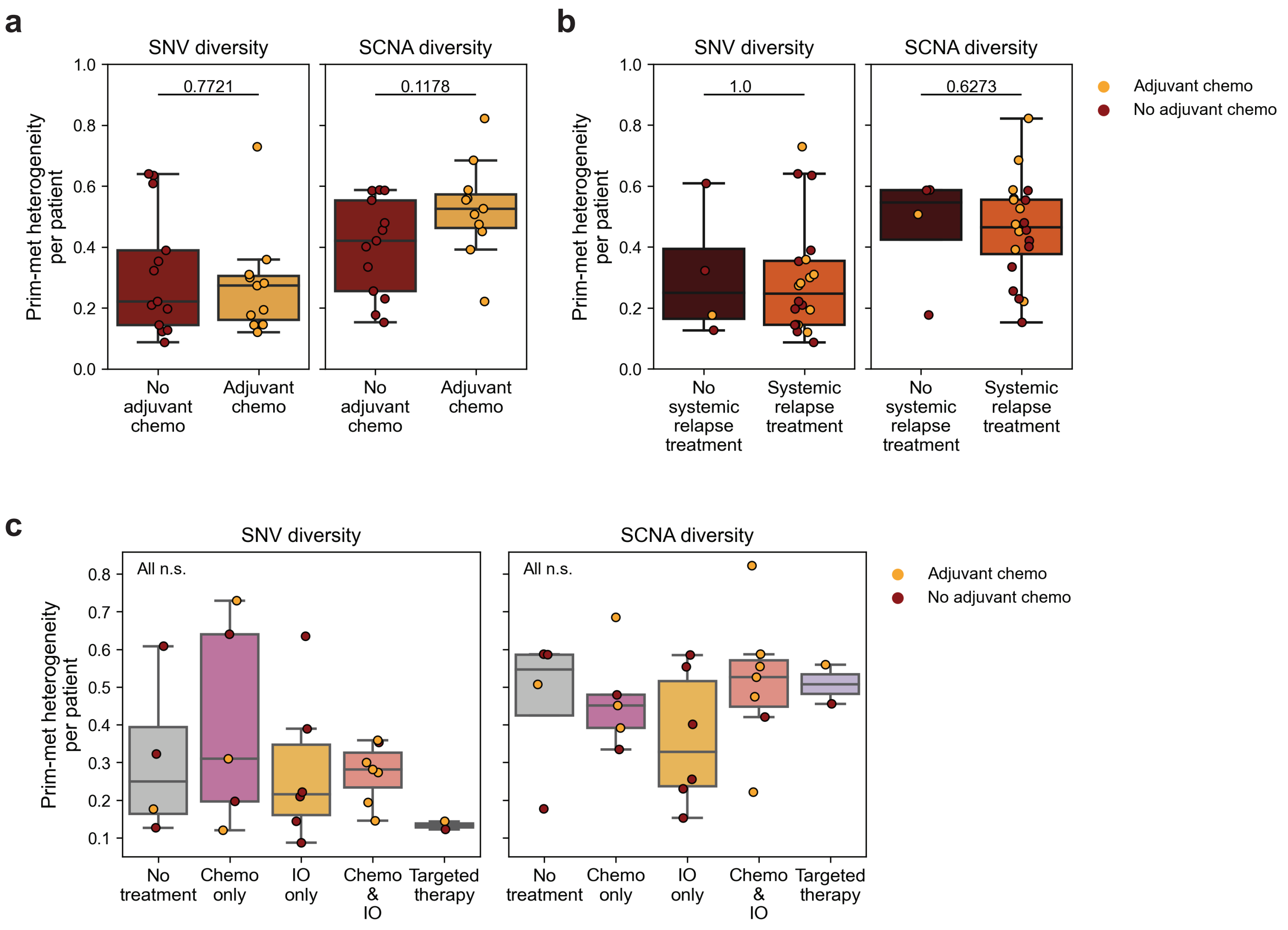

Supplementary Figure 4. Relationship between systemic treatment and primary-metastasis genetic divergence. The mean primary-metastasis SNV and SCNA diversity per patient was compared between patients who did and did not receive adjuvant chemotherapy (**a**), patients who did and did not receive systemic treatment of any kind following relapse with metastatic disease (**b**), and between patients who received different forms of systemic treatment following relapse (**c**). The box plots show the median and IQR with whiskers denoting values within 1.5 times the IQR from the first and third quartiles.

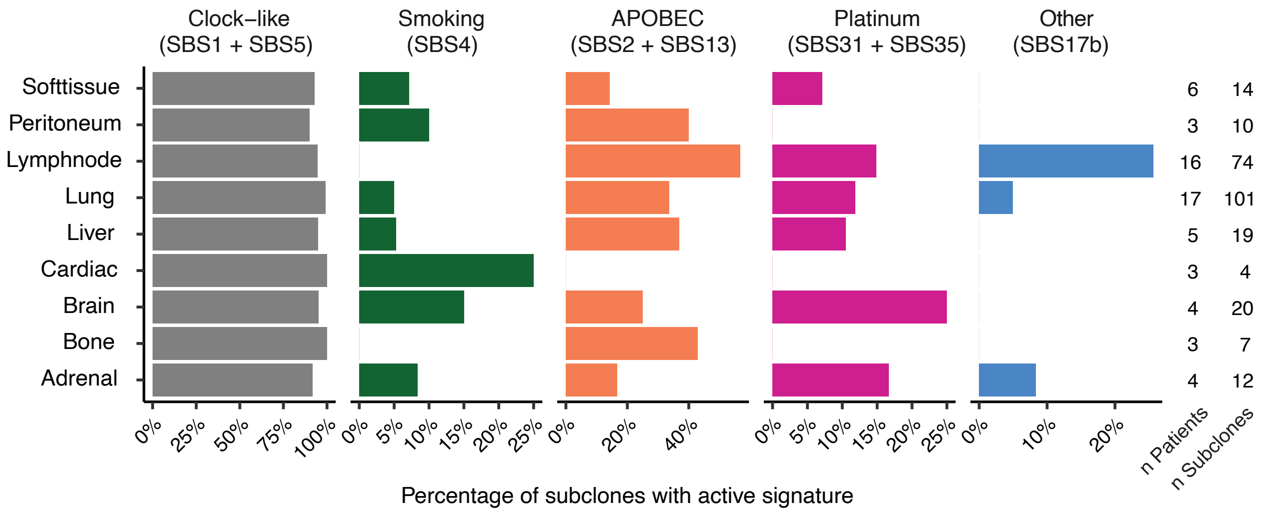

Supplementary Figure 5. Site-specific mutational signature activity. The percentage of metastatic-unique subclones which are unique to a single anatomical location and their signature activities summarized by the prevalence of aetiologies detected above 0.06 estimated activity threshold.

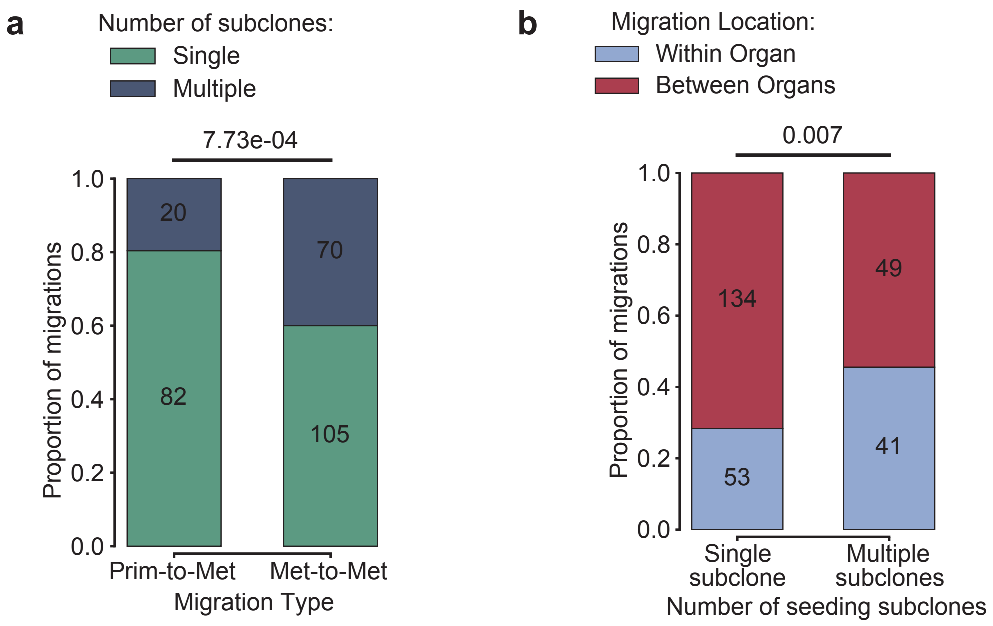

Supplementary Figure 6. The origin and location of migrations involving single or multiple tumour subclones. a, The proportion of primary-to-metastasis and metastasis-to-metastasis migrations that involve the migration of a single subclone or multiple (>1) subclones, Chi-squared test. **b,** The proportion of migrations that involve either a single subclone or multiple (>1) subclones where the source of the migration is within the same organ or in a different organ, Chi-squared test.

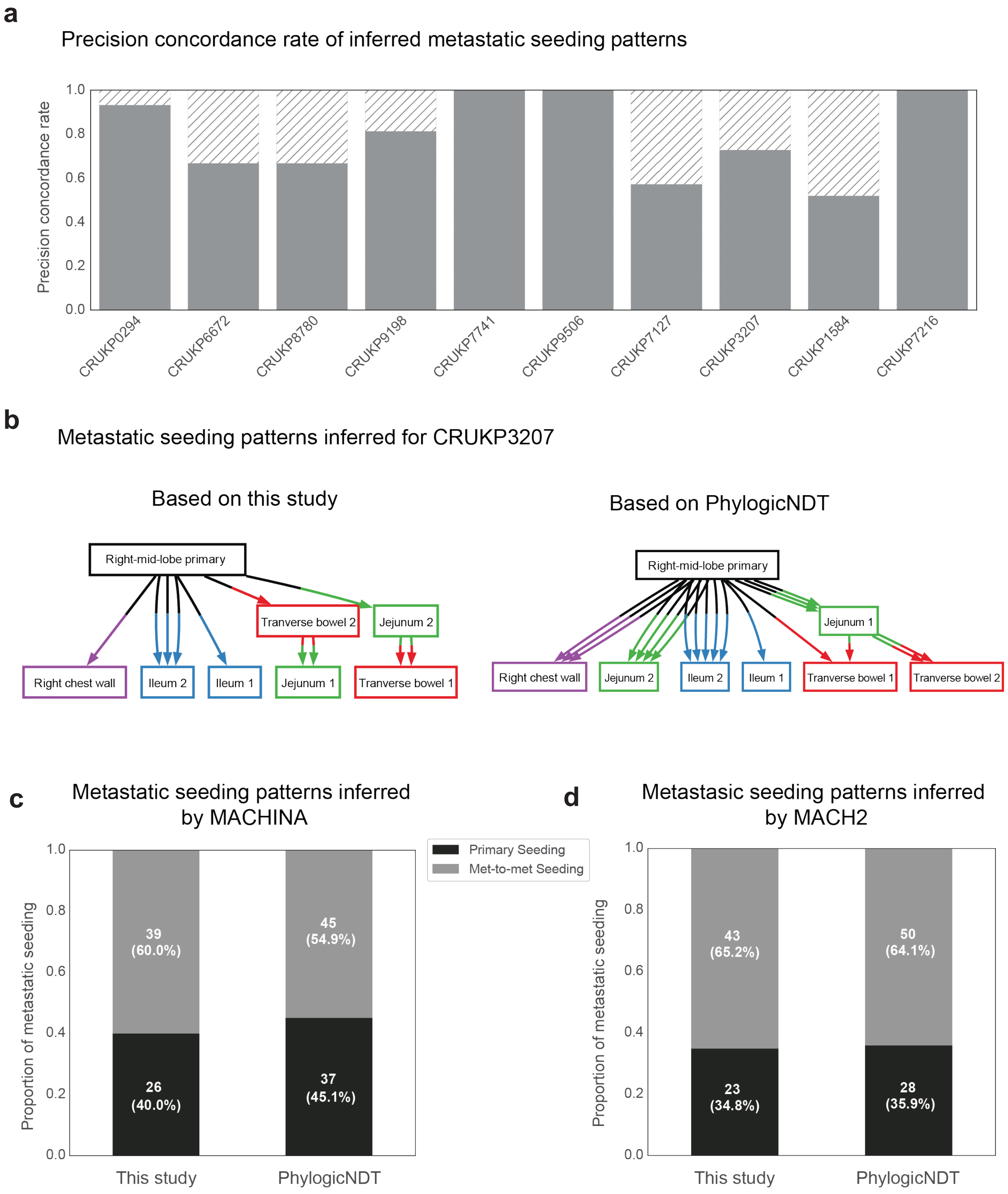

Supplementary Figure 7. Comparison of metastatic seeding patterns inferred using different algorithms. a, The precision concordance rate (y-axis) is calculated for each patient (x-axis) as the fraction of metastatic migrations that are inferred in this study which are also inferred based on the trees inferred by PhylogicNDT. **b,** Example of the metastatic migration patterns inferred by MACHINA from the tree used in this study (left) or inferred by PhylogicNDT (right). **c,** Frequency of primary-to-metastasis vs metastasis-to-metastasis migrations (grey vs black) estimated by MACHINA using either the trees analyzed in this study or those inferred by PhylogicNDT on 10 patients successfully analyzed by PhylogicNDT. **d,** Frequency of primary-to-metastasis (black) vs metastasis-to-metastasis migrations (grey) estimated by MACH2 using the trees either analyzed in this study or inferred by PhylogicNDT on the same patients.

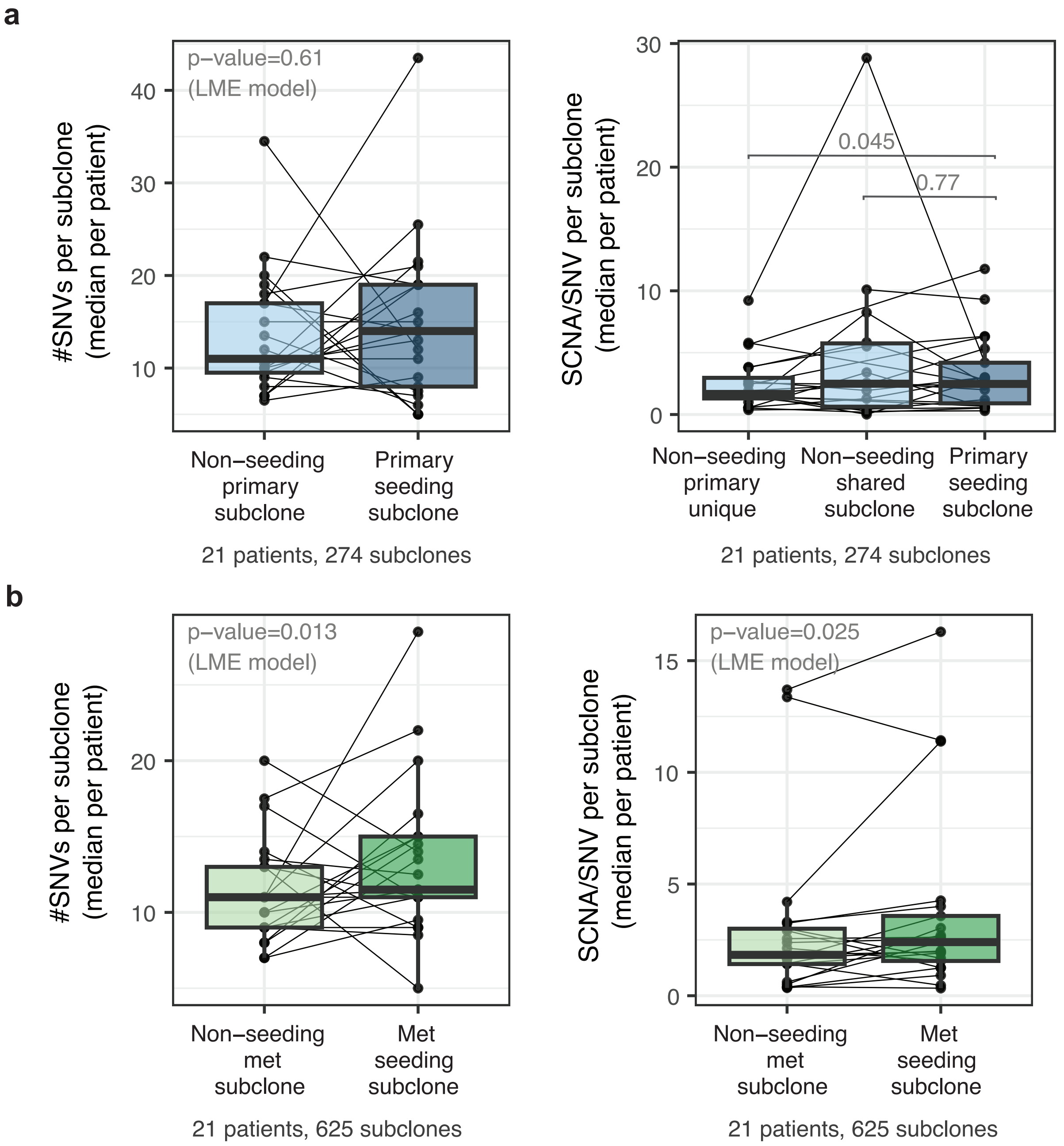

Supplementary Figure 8. SNVs and SCNA acquisition rate in seeding and non-seeding subclones. The number of single nucleotide variants (SNVs, left) and ratio of somatic copy number events (SCNAs) to SNVs (SCNA/SNV, right) per subclone was compared for primary-to-metastasis seeding subclones and primary non-seeding subclones (**a**) and for metastasis-to-metastasis seeding subclones and metastasis non-seeding subclones (**b**). Only tumours with seeding and non-seeding subclones are considered, dots represent the median SCNA rate per patient tumour, lines connect patients. Linear mixed-effects (LME) model with patient as a random effect. The box plots show the median and IQR with whiskers denoting values within 1.5 times the IQR from the first and third quartiles.

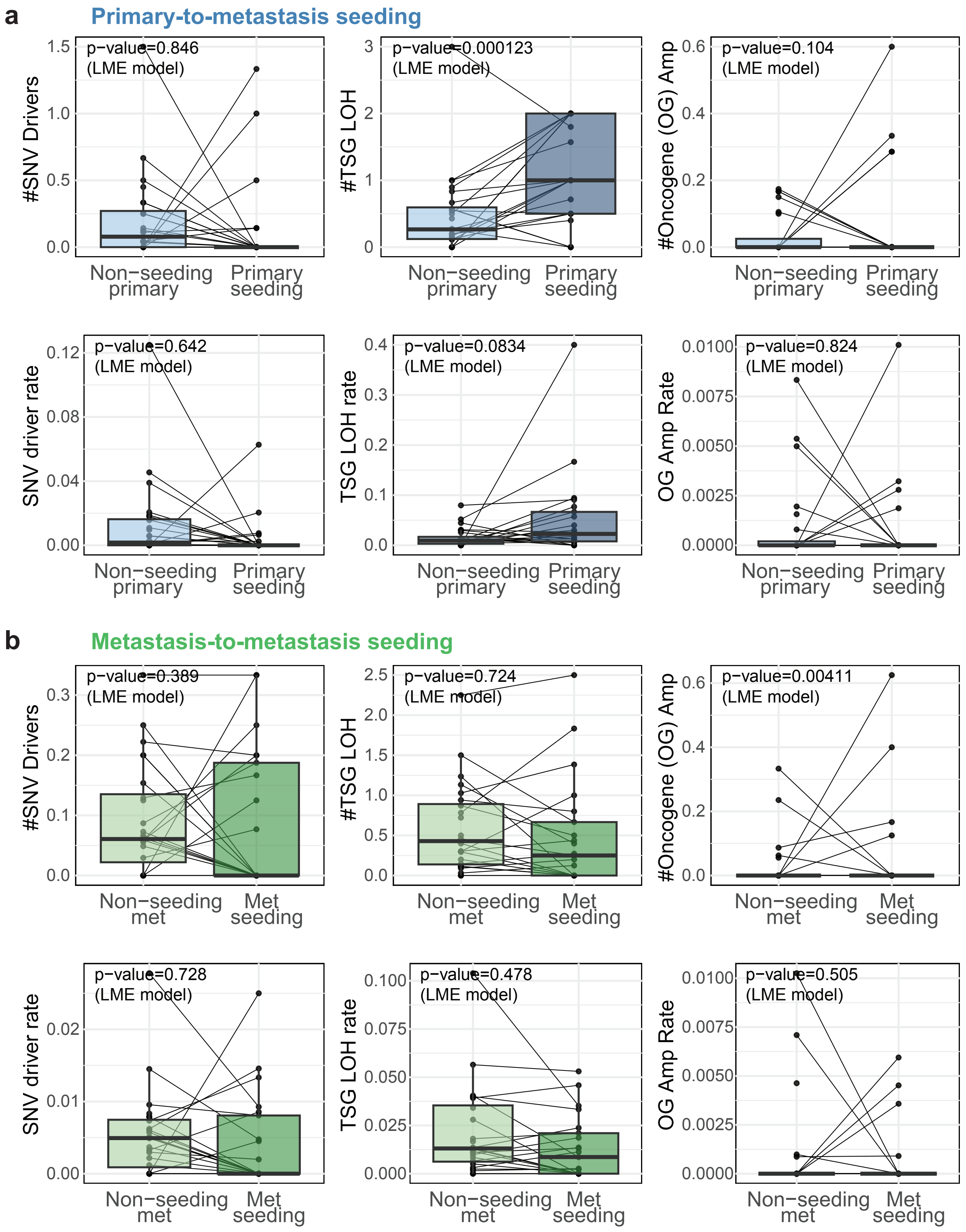

Supplementary Figure 9. Driver alterations and seeding. Comparison of the number and rate (total number normalized by the subclone SNV or SCNA burden) of single nucleotide variant (SNV) drivers, loss of heterozygosity (LOH) affecting tumour suppressor genes (TSGs) or amplifications (Amp) affecting oncogenes (OG) between primary-to-metastasis seeding subclones and non-seeding primary subclones (**a**) and metastasis-to-metastasis seeding subclones and non-seeding metastasis subclones (**b**). Linear mixed-effects (LME) model with patient as a random effect. The box plots show the median and IQR with whiskers denoting values within 1.5 times the IQR from the first and third quartiles.

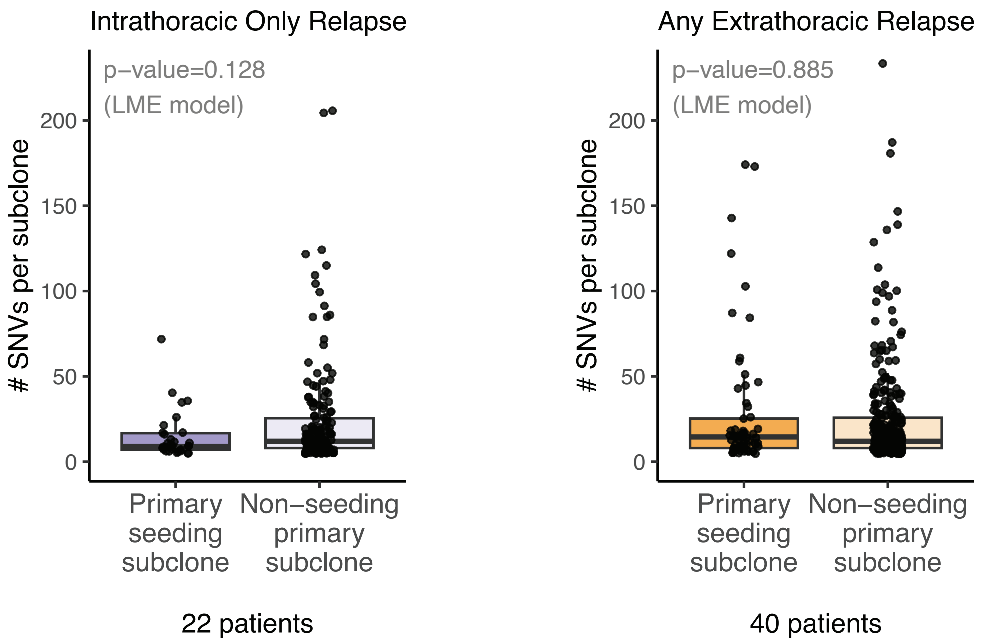

Supplementary Figure 10. SNV burden of seeding and non-seeding subclones in the TRACERx 421 relapse cohort stratified by relapse site. Site of relapse based on radiological imaging was known for 62/126 patients in the published TRACERx 421 relapse cohort. SNV burden of seeding and non-seeding primary subclones was compared for 22 patients with intrathoracic only relapse (left) and 40 patients with 1 or more extrathoracic metastasis at relapse (right). Linear mixed-effects (LME) model with patient as random effect. The box plots show the median and IQR with whiskers denoting values within 1.5 times the IQR from the first and third quartiles.

Supplementary Tables

Characteristic	N = 24[1]
**Age**	70 (63,77)
**Sex**	
Female	14 (58%)
Male	10 (42%)
**Ethnicity**	
White	22 (92%)
Black or Caribbean	2 (8.3%)
**Smoking status**	
Never smoker	2 (9.5%)
Ex-smoker	13 (62%)
Current smoker	6 (29%)
**Pack years**	23 (12, 58)
**Histology**	
LUAD	9 (38%)
LUSC	10 (42%)
Other	5 (21%)
**TNM stage (version 8)**	
IA-B	4 (17%)
IIA-B	8 (33%)
IIIA-B	12 (50%)
**Adjuvant treatment**	13 (54%)
**Adjuvant treatment type**	
Platinum chemotherapy	9 (69%)
Radiotherapy	2 (15%)
Both	2 (15%)
**Number of adjuvant cycles**	
1	2 (18%)
2	2 (18%)
3	2 (18%)
4+	5 (45%)
**Margin status**	
R0	22 (92%)
R1	2 (8%)
**Relapse site (imaging)**	
Intrathoracic	10 (43%)
Extrathoracic	3 (13%)
Intra & Extra	10 (43%)
**DFS (months)**	11 (5,18)
**OS (months)**	29 (15,46)

[1]Median (Q1, Q3); n (%)

Supplementary Table 1. Clinical characteristics of patients in the TRACERx-PEACE Lung cohort. LUAD: lung adenocarcinoma, LUSC: lung squamous cell carcinoma, Other: Non-LUAD or non-LUSC subtype. DFS: disease free survival, OS: overall survival.

Supplementary Notes

1 Anatomical classifications informed by radiological imaging review

Eight patients had chest wall or complex thoracic masses sampled pre-mortem or at autopsy. Chest wall metastases were considered extrathoracic unless radiological imaging evidence was available that suggested the metastasis originated within the visceral pleura and extended into the chest wall. Details of the imaging review that guided the intrathoracic or extrathoracic classification of these metastasis samples are below.

CRUKP3371: Imaging demonstrated metastatic relapse at the site of prior resection that involved the right posterior pleura and posterior chest wall, corresponding most closely to the autopsy sample *right chest wall 1*. Subsequent imaging demonstrated an antero-lateral intercostal space mass between the right 6th and 7th ribs, corresponding to the *anterior chest wall* mass and *right inner costal wall* masses sampled at autopsy. On the last imaging performed prior to death these chest wall masses were indistinguishable from disease within the right lung and hilum. Thus, all were classified as intrathoracic.

CRUKP8780: Imaging demonstrated multiple right lower lobe lung metastases and multifocal cystic lesions in the right lower hemithorax that indented the diaphragm and extended to the skin. This complex pulmonary-paramediastinal mass corresponds to the *subcutaneous chest wall mass* sampled at autopsy which was classified as intrathoracic.

CRUKP3207: A chest wall nodule was clinically identified in the thoracotomy wound. No associated mediastinal lymph nodes, pleural disease or effusion or pulmonary metastases were detected on relapse or follow up imaging. This most closely corresponds to the sampled *right chest wall 2* metastasis classified as extrathoracic.

CRUKP8172: This patient required pleurodesis for a post-operative hydropneumothorax, complicating interpretation of pleural appearances on imaging. A left pleural biopsy confirmed relapse adjacent to the primary resection site. Metastases affecting the left posterior 3rd, 4th and 6th ribs, the right 5th rib, and T11 vertebrae associated with adjacent soft tissue extension were detected on imaging performed shortly after relapse. The chest wall masses sampled at autopsy were most closely related to these lesions and were classified as extrathoracic. Bilateral lung metastases were also detected on imaging. The mediastinal soft tissue mass seen on imaging performed prior to death, attributed to the *ascending aorta nodule* sampled at autopsy, was classified as intrathoracic.

CRUKP8433: The imaging detected focal area of nodularity between the 3rd and 4th rib associated with subpectoral and axillary lymphadenopathy, corresponding to the *intercostal chest wall mass* sampled at autopsy, was classified as extrathoracic.

CRUKP4761: Relapse imaging demonstrated a metastasis in the T8/9 intervertebral disc space. The T8/9 *paravertebral mass* sampled at autopsy was classified as extrathoracic. Subsequent imaging demonstrated a further, adjacent, enlarging paraspinal soft tissue mass in communication with the mediastinum corresponding to the *paraaortic mass 1* and *2* samples which were classified as intrathoracic.

CRUKP2432: Imaging identified a soft tissue lesion arising from the posteriomedial pleural surface and two other pleural metastases in the right lung. Later imaging identified further right sided pleural disease invading the anterior mediastinum, extending into the apex and infiltrating through the chest wall anteriorly, involving the pectoralis muscles. The tumour crossed the midline and extended to the left side of the mediastinum and abutted much of the right hemidiaphragm. Several samples were taken of this large complex mass at autopsy, all classified as intrathoracic.
