# Supplementary material for: Evolutionary characterization of lung cancer metastasis
Source: Nature. 2026 Apr 29;653(8115):911–22. doi: 10.1038/s41586-026-10428-4 (PMC13190308; doi:10.1038/s41586-026-10428-4)
Supplement: Supplementary file 2 — Reporting Summary [file 41586_2026_10428_MOESM2_ESM.pdf]

nature portfolio

Corresponding author(s): Mariam Jamal-Hanjani
Nicholas McGranahan
Charles Swanton

Last updated by author(s): Feb 4, 2026

Reporting Summary

Nature Portfolio wishes to improve the reproducibility of the work that we publish. This form provides structure for consistency and transparency in reporting. For further information on Nature Portfolio policies, see our Editorial Policies and the Editorial Policy Checklist.

Statistics

For all statistical analyses, confirm that the following items are present in the figure legend, table legend, main text, or Methods section.

n/a	Confirmed	
☐	☒	The exact sample size (*n*) for each experimental group/condition, given as a discrete number and unit of measurement
☐	☒	A statement on whether measurements were taken from distinct samples or whether the same sample was measured repeatedly
☐	☒	The statistical test(s) used AND whether they are one- or two-sided<br>*Only common tests should be described solely by name; describe more complex techniques in the Methods section.*
☐	☒	A description of all covariates tested
☐	☒	A description of any assumptions or corrections, such as tests of normality and adjustment for multiple comparisons
☐	☒	A full description of the statistical parameters including central tendency (e.g. means) or other basic estimates (e.g. regression coefficient) AND variation (e.g. standard deviation) or associated estimates of uncertainty (e.g. confidence intervals)
☐	☒	For null hypothesis testing, the test statistic (e.g. *F*, *t*, *r*) with confidence intervals, effect sizes, degrees of freedom and *P* value noted<br>*Give P values as exact values whenever suitable.*
☒	☐	For Bayesian analysis, information on the choice of priors and Markov chain Monte Carlo settings
☒	☐	For hierarchical and complex designs, identification of the appropriate level for tests and full reporting of outcomes
☐	☒	Estimates of effect sizes (e.g. Cohen's *d*, Pearson's *r*), indicating how they were calculated

Our web collection on statistics for biologists contains articles on many of the points above.

Software and code

Policy information about availability of computer code

Data collection	No software was used to collect data
Data analysis	The code for the alignment pipeline and the subsequent processing pipeline can be found on github (https://github.com/FrancisCrickInstitute/peace-alignment) and (https://github.com/FrancisCrickInstitute/peace-pipeline), respectively. All code to reproduce analyses and figures is available at https://doi.org/10.5281/zenodo.15755949

For manuscripts utilizing custom algorithms or software that are central to the research but not yet described in published literature, software must be made available to editors and reviewers. We strongly encourage code deposition in a community repository (e.g. GitHub). See the Nature Portfolio guidelines for submitting code & software for further information.

Data

Policy information about availability of data

All manuscripts must include a data availability statement. This statement should provide the following information, where applicable:

- Accession codes, unique identifiers, or web links for publicly available datasets
- A description of any restrictions on data availability
- For clinical datasets or third party data, please ensure that the statement adheres to our policy

The whole exome sequencing data collected from the TRACERx study and PEACE study used in this manuscript has been deposited at the European Genome–Phenome Archive (EGA), which is hosted by the European Bioinformatics Institute (EBI) and the Centre for Genomic Regulation (CRG) under accession code EGAS00001008217 and the associated dataset EGAD00001015763; access is controlled by the PEACE data access committee.

Field-specific reporting

Please select the one below that is the best fit for your research. If you are not sure, read the appropriate sections before making your selection.

☒ Life sciences ☐ Behavioural & social sciences ☐ Ecological, evolutionary & environmental sciences

For a reference copy of the document with all sections, see nature.com/documents/nr-reporting-summary-flat.pdf

Life sciences study design

All studies must disclose on these points even when the disclosure is negative.

Sample size

This manuscript focuses on 24 patients with non-small cell lung cancer (NSCLC) enrolled in the TRACERx lung study and the PEACE study, which is an autopsy programme.
501 tumour regions were sampled longitudinally, encompassing 108 primary tumour regions, 41 regions from metastases sampled pre-mortem and 352 regions from metastases sampled at autopsy.

Data exclusions

Patients were recruited to the TRACERx study according to the inclusion and exclusion criteria detailed in Reporting Summary of https://doi.org/10.1038/s41586-023-05783-5.

Patients were recruited to the PEACE study according to the following inclusion and exclusion criteria:
Inclusion criteria
- Age 18 years or older
- Confirmed diagnosis of any form of solid malignancy with metastatic disease (where the site of origin is known or unknown), with the exception of primary brain tumour in which there may not be evidence of metastatic disease
- Oral and written informed consent from patient to enter the study and to undergo tissue harvesting after death or informed consent from a nominated representative or a person in a qualifying relationship after the patient has died.

Exclusion criteria
- Medical or psychiatric condition that would preclude informed consent
- History of intravenous drug abuse within the last 5 years
- Confirmed diagnosis for high-risk infections (e.g. HIV/AIDS-positive, hepatitis B/C, tuberculosis and Creutzfeldt-Jacob disease) unless patient case is of a particular scientific interest and agreed in advance with local mortuary staff and pathologist.

Supplementary Figure 1 contains a flow chart that details the samples taken forward for sequencing and the samples of sufficient quality to be included in downstream analyses.

Replication

The TRACERx study and PEACE study are prospective, longitudinal, observational studies. As such, the results in this manuscript are not the result of an experimental set up.

Randomization

Not applicable in these observational studies.

Blinding

Not applicable in these observational studies.
Patients were not allocated to an intervention and were followed up according to routine clinical practice.
Molecular profiling results were not fed back to patients. As such, there is no risk in this information influencing their behaviours or outcomes.

Reporting for specific materials, systems and methods

We require information from authors about some types of materials, experimental systems and methods used in many studies. Here, indicate whether each material, system or method listed is relevant to your study. If you are not sure if a list item applies to your research, read the appropriate section before selecting a response.

Materials & experimental systems

n/a	Involved in the study	
☒	☐	Antibodies
☒	☐	Eukaryotic cell lines
☒	☐	Palaeontology and archaeology
☒	☐	Animals and other organisms
☐	☒	Human research participants
☐	☒	Clinical data
☒	☐	Dual use research of concern

Methods

n/a	Involved in the study	
☒	☐	ChIP-seq
☒	☐	Flow cytometry
☒	☐	MRI-based neuroimaging

Human research participants

Policy information about studies involving human research participants

Population characteristics	Supplementary Table 1 summarises the clinical and demographic information for all 24 patients included in this study.
Recruitment	For the TRACERx study, when patients are initially diagnosed with stage I-III lung cancer and then referred for surgical resection, a research nurse identifies them on a clinic/operating list. The patient has an initial eligibility assessment and is then provided with written information about the TRACERx study and the contact details of the research nurse/practitioner to ask any questions.<br><br>For the PEACE study, patients with metastatic disease are approached sensitively during routine clinic appointments or during inpatient hospital admissions (where appropriate) about their interest in participating in a research autopsy programme. If interested, they are provided with written information about the PEACE study and the contact details of the research nurse/practitioner to ask any questions. Notification of a PEACE participant's death is performed by family members or individuals in a qualifying relationship who are also provided the contact details of the research nurse/practitioner.
Ethics oversight	The TRACERx study was approved by the NRES Committee London with the following details:<br>Study title: TRAcking non small cell lung Cancer Evolution through therapy (Rx)<br>REC reference: 13/LO/1546<br>Protocol number: UCL/12/0279<br>IRAS project ID: 138871<br><br>The PEACE study was approved by the NRES Committee London with the following details:<br>Study title: PEACE (Posthumous Evaluation of Advanced Cancer Environment) Study<br>REC reference: 13/LO/0972<br>Protocol number: UCL/13/0165<br>IRAS project ID: 125424<br><br>Written informed consent was obtained from all participants (separately for the TRACERx study and the PEACE study).

Note that full information on the approval of the study protocol must also be provided in the manuscript.

Clinical data

Policy information about clinical studies

All manuscripts should comply with the ICMJE guidelines for publication of clinical research and a completed CONSORT checklist must be included with all submissions.

Clinical trial registration	TRAcking non small cell lung Cancer Evolution through therapy (Rx); NCT01888601<br>PEACE (Posthumous Evaluation of Advanced Cancer Environment) Study; NCT03004755
Study protocol	https://clinicaltrials.gov/study/NCT01888601<br>https://clinicaltrials.gov/study/NCT03004755
Data collection	Clinical and pathological data were collected from the patients from the time of they enrolled in the TRACERx study through to death in accordance with the TRACERx study protocol. Data collection was overseen by the sponsor of the study (Cancer Research UK & UCL Cancer Trials Centre) and takes place in hospitals across the United Kingdom. A centralised database called MACRO is used for this purpose.
Outcomes	The pre-defined outcome measures for the TRACERx study are detailed in the Reporting Summary of https://doi.org/10.1038/s41586-023-05783-5.<br>By enabling extensive tumour sampling at the time of death, the PEACE study builds on the longitudinal samples collected through the TRACERx study for patients who participated in both studies. The analyses conducted in this manuscript are hypothesis generating.
