# Supplementary material for: Evolutionary characterization of lung cancer metastasis
Source: Nature. 2026 Apr 29;653(8115):911–22. doi: 10.1038/s41586-026-10428-4 (PMC13190308; doi:10.1038/s41586-026-10428-4)
Supplement: Supplementary file 3 — PEACE protocol [file 41586_2026_10428_MOESM3_ESM.pdf]

Docusign Envelope ID: AB97AAF1-C9D6-4C1D-9E5C-84B38883CE23

PEACE

The PEACE (Posthumous Evaluation of Advanced Cancer Environment) Study

Study Sponsor:	University College London
Study Sponsor reference:	13/0165
Study funder:	Cancer Research UK Centre Accelerator Award
Funder reference:	C416/A21999
Study REC reference:	13/LO/0972
NIHR portfolio reference:	18422
Protocol version no:	9.1
Protocol version date:	11/09/2024

Protocol 9.1 – 11/09/2024 Authorisation signatures

Name & Role:	Signature:	Date authorised:
Chief Investigator:		
Professor Mariam Jamal-Hanjani<br>Professor of Cancer Genomics and Metastasis	Signed by:<br>Mariam Jamal-Hanjani<br>8717E72CDF6B4F0...	23-Sep-2024
Aoife Walker<br>Senior Project Manager<br>CRUK and UCL Cancer Trials Centre	DocuSigned by:<br>Aoife Walker<br>3CBDD04C3FF548F...	23-Sep-2024

Please note: This Study protocol must not be applied to patients outside the PEACE Study. UCL CTC can only ensure that approved Study Investigators are provided with amendments to the protocol.

Docusign Envelope ID: AB97AAF1-C9D6-4C1D-9E5C-84B38883CE23

COORDINATING CENTRE:

For general queries, supply of Study documentation and central data management please contact:

PEACE Trial Manager
Cancer Research UK & UCL Cancer Trial Centre
90 Tottenham Court Road
London
W1T 4TJ

Tel: +44 (0) 20 7679 9016
09:00 to 17:00 Monday to Friday, excluding Bank Holidays (UK time)

Email: ctc.peace@ucl.ac.uk

Other Study contacts:

Chief Investigator: Professor Mariam Jamal-Hanjani
Address: Paul O'Gorman Building
UCL Cancer Institute
London
WC1E 6BT

PEACE Consortium

Nb. The consortium list will be updated as applicable. Please contact UCL CTC for a current list.

Docusign Envelope ID: AB97AAF1-C9D6-4C1D-9E5C-84B38883CE23

TABLE OF CONTENTS

1. PROTOCOL SUMMARY 5
1.1. SUMMARY OF STUDY DESIGN 5
2. INTRODUCTION 11
2.1. BACKGROUND 11
2.2. DEFINING THE ORIGINS OF THE LETHAL SUBCLONE(S) IN CANCER 11
2.3. INSIGHT INTO TUMOUR BIOLOGY AND EVOLUTION USING CIRCULATING BIOMARKERS 12
2.4. INTRATUMOUR HETEROGENEITY AND THE TUMOUR NEO-ANTIGEN REPERTOIRE 13
2.5. CANCER OF UNKNOWN PRIMARY: IDENTIFYING TUMOUR ORIGIN AND MODEL OF EARLY METASTASIS 13
2.6. CREATING A DIGITAL ARCHIVE FOR SAMPLES 14
3. STUDY DESIGN 15
3.1. STUDY OBJECTIVES 15
3.2. STUDY ACTIVATION 15
4. SELECTION OF SITES/SITE INVESTIGATORS 17
4.1. SITE SELECTION 17
4.1.1. Selection of Principal Investigator and other investigators at sites 17
4.1.2. Training requirements for site staff 17
4.2. SITE INITIATION AND ACTIVATION 17
4.2.1. Site initiation 17
4.2.2. Required documentation 18
4.2.3. Site activation letter 18
5. INFORMED CONSENT 19
6. SELECTION OF PATIENTS 23
PRE-REGISTRATION EVALUATION 23
6.1. SCREENING LOG 23
6.2. PATIENT ELIGIBILITY 23
6.2.1. Inclusion criteria 24
6.2.2. Exclusion criteria 24
6.2.3. Recruitment Selection 24
7. REGISTRATION PROCEDURES 25
7.1. REGISTRATION 25
8. STUDY SPECIFIC PROCEDURES 26
8.1. BLOOD SAMPLE COLLECTION 26
8.2. IMAGING PRIOR TO TISSUE SAMPLING 27
8.3. BIOLOGICAL TISSUE SAMPLES 27
8.4. PLANNED SAMPLE ANALYSIS 30
8.5. CREATING A CENTRAL DIGITAL ARCHIVE 32
8.6. COLLECTION OF CLINICOPATHOLOGICAL AND DEMOGRAPHIC DATA 32
9. DATA MANAGEMENT AND DATA HANDLING GUIDELINES 33
9.1. DATA ENTRY 33
9.2. MISSING DATA 33
9.3. TIMELINES FOR DATA ENTRY 33
9.4. DATA QUERIES 33
10. INCIDENT REPORTING, STUDY MONITORING AND OVERSIGHT 34

Docusign Envelope ID: AB97AAF1-C9D6-4C1D-9E5C-84B38883CE23

10.1. INCIDENT REPORTING 34
10.2. ON-SITE MONITORING 34
10.3. CENTRALISED MONITORING 34
10.4. 'TRIGGERED' ON-SITE MONITORING 35
10.5. OVERSIGHT COMMITTEES 35
10.5.1. PEACE Consortium 35
10.5.2. Study Governance Board (SGB) 35
10.5.3. Independent Data Monitoring Committee (IDMC) 36
10.5.4. Role of UCL CTC 36

11. WITHDRAWAL OF PATIENTS 37

11.1. DISCONTINUATION OF STUDY PARTICIPATION 37
11.2. FUTURE DATA COLLECTION 37
11.3. LOSSES TO FOLLOW-UP 37

12. STUDY CLOSURE 38

12.1. END OF STUDY 38
12.2. ARCHIVING OF STUDY DOCUMENTATION 38
12.3. EARLY DISCONTINUATION OF STUDY 38
12.4. WITHDRAWAL FROM STUDY PARTICIPATION BY A SITE 38

13. QUALITY ASSURANCE 39

14. ETHICAL APPROVALS 40

14.1. ETHICAL APPROVAL 40
14.2. SPECIFIC ETHICAL ISSUES 40
14.3. SITE APPROVALS 41
14.4. PROTOCOL AMENDMENTS 41
14.5. PATIENT CONFIDENTIALITY & DATA PROTECTION 41

15. SPONSORSHIP AND INDEMNITY 42

15.1. SPONSOR DETAILS 42
15.2. INDEMNITY 42

16. FUNDING 43

17. PUBLICATION POLICY 44

18. REFERENCES 45

APPENDIX 1: ABBREVIATIONS 48

APPENDIX 2: PROTOCOL VERSION HISTORY 50

Docusign Envelope ID: AB97AAF1-C9D6-4C1D-9E5C-84B38883CE23

1. PROTOCOL SUMMARY

1.1. Summary of Study Design

**Title:**	Posthumous Evaluation of Advanced Cancer Environment Study
**Short Title/acronym:**	PEACE
**Sponsor name & reference:**	University College London<br>Ref: 13/0165
**Funder name & reference:**	Cancer Research UK Centre Accelerator Award. Various dedicated funders as PEACE incorporates specific research questions with dedicated funding that leverages the scientific and research infrastructure.
**Design:**	PEACE is a multi-centre prospective observational study
**Overall aim:**	To facilitate tissue donation from multiple tumour sites in the post-mortem setting
**Additional Aims**	To recruit patients in existing clinical studies involving tissue collection and in-depth genomics analyses with high-quality clinical data collection<br>To recruit patients of particular research interest and potential for in-depth analyses, such as those with primary tumours *in situ*
**Target accrual:**	800
**Inclusion & exclusion criteria:**	**Inclusion criteria**<br>• Age 18 years or older<br>• Confirmed diagnosis of any form of solid malignancy with metastatic disease (where the site of origin is known or unknown), with the exception of primary brain tumour in which there may not be evidence of metastatic disease<br>• Oral and written informed consent from patient to enter the study and to undergo tissue harvesting after death or informed consent from a nominated representative or a person in a qualifying relationship after the patient has died.

Docusign Envelope ID: AB97AAF1-C9D6-4C1D-9E5C-84B38883CE23

	**Exclusion Criteria**<br>• Medical or psychiatric condition that would preclude informed consent<br>• History of intravenous drug abuse within the last 5 years<br>• Confirmed diagnosis of known high-risk infections (e.g. HIV/AIDS-positive, hepatitis B/C, tuberculosis and Creutzfeldt-Jacob disease) unless patient case is of a particular scientific interest and agreed in advance with local mortuary staff and pathologist.
**Planned number of sites:**	Approximately 10-15
**Central laboratories receiving samples**	UCL Cancer Institute (University College London), CEP GCLP Laboratories (University of Manchester).
**Collaborating translational research laboratories**	The Francis Crick Institute, UCL Cancer Institute, University of Leicester, University of Cambridge, The Broad Institute, University of Groningen, Genomics England, Institute of Cancer Research, Imperial College London, University of Manchester, Manchester Cancer Research Centre, Lungs for Living Research Centre, The Royal National Orthopaedic Hospital, Leicester Royal Infirmary, The Christie NHS Foundation Trust, Wellcome Sanger Institute, University College London Hospital, John Vance Science Centre, Queen Mary University of London, SAGA Diagnostics, Brain Metastasis Group Molecular Oncology Programme, CNIO, Leeds Institution of Cardiovascular & Metabolic Medicine
**Summary of procedures:**	Blood samples will be taken at baseline, subsequent follow up visits and after death for germline DNA (baseline only and after death only), cfDNA, CTC and immunological analyses (bloods for immunological analysis are only required for patients who are also participating in TRACERx EVO).<br><br>Tissue harvesting will be performed after death. Tissue will be collected from sites of tumour and normal tissue, guided by imaging, findings at tissue harvest or clinical history. This will entail:

Docusign Envelope ID: AB97AAF1-C9D6-4C1D-9E5C-84B38883CE23

	• Sampling of all tumour tissue (this may involve the removal of organs to allow sampling)<br>• Extensive background sampling of normal tissue in diseased organs (in the case of prostate and brain cancer this may involve sampling of the entire prostate/brain)<br>• Smaller scale sampling of normal tissue<br><br>The following samples may also be collected: cerebrospinal fluid, pleural fluid, pericardial fluid, peritoneal fluid, urine and bone marrow.<br>Any remaining tissue will be returned to the body.
**Anticipated duration of recruitment:**	Recruitment timelines are dependent on the recruitment and subsequent death of up to 100 patients on the TRACERx EVO study but recruitment will close 2030
**Definition of end of Study:**	End of study will be 2030
**Translational component:**	The PEACE study is intended to enable future research using samples collected at tissue harvests in a post-mortem setting within different disciplines related to cancer research. Furthermore, preference will be for the study to complement existing or planned studies involving tissue collection and in-depth genomics analyses with high-quality clinical data collection, such as the TRACERx EVO study.<br><br>Patient recruitment will be within the context of PEACE-specific research proposals approved by the Study Governance Board. From October 2024, new further research will be overseen and approved by the Study Oversight Group.
**Other related research:**	The PEACE study will enable research in areas such as:<br>• Evolution of cancer and its microenvironment/immune landscape<br>• Mechanisms of drug resistance<br>• Clinical utility of circulating biomarkers<br>• Predictive and prognostic biomarkers<br>• Role of cancer metabolism in metastatic disease

Docusign Envelope ID: AB97AAF1-C9D6-4C1D-9E5C-84B38883CE23

	• Multiomic landscape of background normal tissue<br>• Defining the origins of the lethal subclone(s) in cancer<br>• Insight into tumour biology and evolution using circulating biomarkers<br>• Intratumour heterogeneity and the tumour neo-antigen repertoire<br>• Cancer of unknown primary: identifying tumour origin and model of early metastasis<br>• Creating a digital pathology archive

Docusign Envelope ID: AB97AAF1-C9D6-4C1D-9E5C-84B38883CE23

Study Pathway

Generic PEACE study patient pathway (if participant dies in a PEACE participating hospital) This pathway can be adapted for your institution – for guidance only

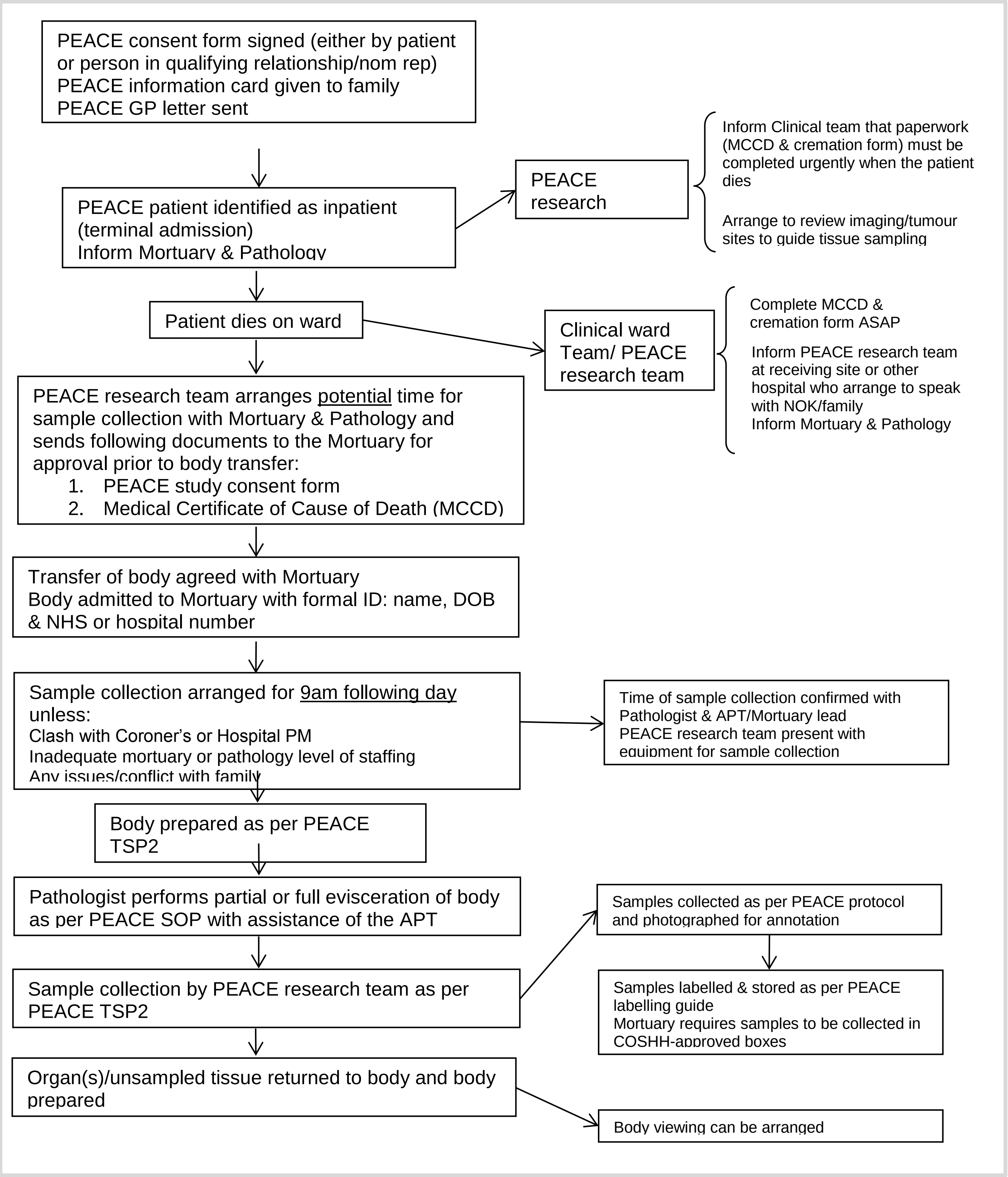

Generic PEACE study patient pathway (if participant DOES NOT die in a PEACE participating hospital, – participant dies in non-PEACE hospital, hospice or home)

This pathway can be adapted for your institution – for guidance only

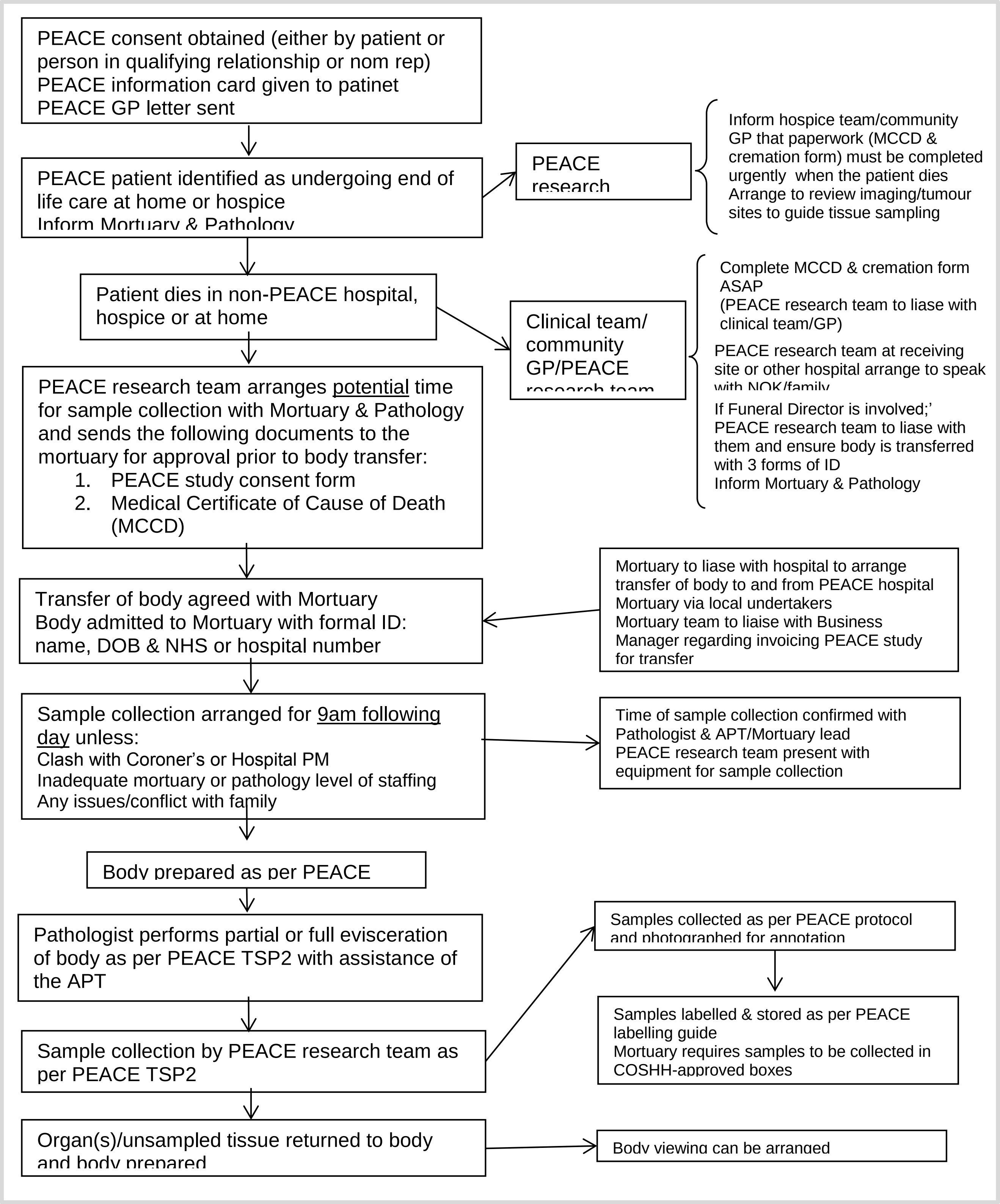

Docusign Envelope ID: AB97AAF1-C9D6-4C1D-9E5C-84B38883CE23

2. INTRODUCTION

2.1. Background

Despite recent advances in targeted therapy, the molecular understanding of cancer and the evolution of metastasis and resistance to therapy is limited. Studies of tumour pathogenesis and cancer evolution have historically relied on archival tissue that is surplus to diagnostic requirements donated from living patients. These samples have often been obtained at time points relating to cancer diagnosis (often biased towards early stage disease), or less commonly relapsed or drug resistant disease. Spatial and temporal tumour genetic heterogeneity results in significant challenges to the effective understanding of the cancer genome landscape and its evolutionary history.

There are several challenges in obtaining tissue from patients with advanced cancer including the increased biopsy risk associated with multiple site sampling, the technical difficulty in accessing sites of metastatic disease and the inevitable time, travel and costs incurred by patients who are often unwell with a poor performance status. Most patients with widely disseminated disease do not undergo tumour resection, and therefore the ability to obtain sufficient tissue for research purposes is often limited. Potentially less invasive core biopsies are a possibility in this group of patients, but would not be representative of the entire tumour landscape or disease burden. The general lack of primary and metastatic tumour samples has meant that cancer research has often been restricted to either established tumour cell lines or archival material[1] limiting the systematic study of the metastatic process[2]. There are few cancer studies in which post-mortem tumour harvesting is performed, including, but not limited to, studies in breast[3,4], prostate[1,5], pancreatic[2], and uveal melanoma[6] cancers. None of these studies have explored in detail the genetic and phenotypic aberrations and biological pathways involved in the metastatic process, and have often involved small patient cohorts with limited clinical histories. Access to post-mortem tissue can facilitate the in-depth study of the genetic and phenotypic relationships between primary and metastatic tumours with the intention of establishing a model for tumour progression, and therefore the metastatic process. Furthermore, studies of such genetic and phenotypic diversity may help determine the relationship between such diversity and clinical outcome, including response and resistance to treatment and survival.

The PEACE study is intended to enable future research using samples collected at tissue harvests in a post-mortem setting within different disciplines related to cancer research Furthermore, the study will complement existing or planned studies involving tissue collection and in-depth genomics analyses with high-quality clinical data collection, such as the TRACERx and TRACERx EVO national cancer programmes.

The background to some of the potential future research projects that can be enabled by the PEACE study is discussed below.

2.2. Defining the origins of the lethal subclone(s) in cancer

Understanding the evolutionary genetic and epigenetic landscape of tumours will provide key knowledge of potential driver events and help to identify novel therapeutic targets

Docusign Envelope ID: AB97AAF1-C9D6-4C1D-9E5C-84B38883CE23

implicated in metastatic outgrowth and drug resistance in cancer. Clonal diversity between primary and metastatic tumours in the same patient has been demonstrated in different tumour types, including renal[8], lung[9], breast[10], pancreatic[11,12], colorectal[13,14], gastric[15], prostate[16-18] and medulloblastoma[19] tumours. This has also been demonstrated within the same site of disease in primary brain tumours[20-23]. The dynamics and patterns of clonal composition can indicate the tumour evolutionary paths that underlie tumour progression, including potential mechanisms involved in therapeutic resistance and branched tumour evolution[24-26]. Using whole-genome sequencing and whole-exome sequencing, Juric and colleagues sequenced the primary and metastatic tumours in a breast cancer patient with a known activating clonal *PIK3CA* mutation who had developed drug resistance whilst on treatment with a PIK3CA inhibitor[3]. All the metastatic tumours were found to have a common copy number loss of *PTEN*, and those metastatic tumours that were identified as drug resistant based on imaging, were found to have acquired additional convergent genetic aberrations in *PTEN* resulting in the loss of its expression, including SNVs, indels and copy number aberrations. This demonstrated parallel evolution in separate metastatic tumours within the same patient with different aberrations in *PTEN* leading to a convergent phenotype resistant to PIK3CA inhibition. This study provides compelling evidence of the polygenic and convergent nature of drug resistance and important insight into the acquisition of resistance to targeted therapies. In a case of prostate cancer in a patient who presented 17-years after diagnosis with metastatic disease, Haffner and colleagues used whole-genome sequencing and molecular pathological analyses to identify and track the evolution of the lethal clone from the primary tumour to the metastases, and demonstrated that surprisingly this clone originated from a low-grade focus, as opposed to the high-grade bulk, of the primary tumour or the subsequent lymph node metastasis[16]. These studies demonstrate that by mapping the evolution of tumours from the primary to different loco-regional or metastatic sites, the lethal subclone(s) harbouring candidate driver mutations resulting in disease progression, therapeutic resistance, and subsequent overwhelming disease burden and death, can be identified. Access to post-mortem tissue, in particular multi-region sampling within larger tumours and sampling of all sites of metastatic disease, can help define these relationships in order to establish models of metastatic disease and tumour evolution.

2.3. Insight into tumour biology and evolution using circulating biomarkers

Several studies have demonstrated that genetic aberrations present in tumours can be detected in cfDNA, and that these can be used to track tumour evolution, as well as the mutational burden, in patients over time[27-36]. CTCs have also been used to study mutations and SCNAs, and have been shown to correlate with clinical outcome[37-41]. These studies have demonstrated the potential in using circulating biomarkers to track tumour evolution and help guide treatment stratification. The collection of circulating biomarkers in the PEACE study from patients with metastatic cancer and primary brain tumours, will help to determine the extent to which such biomarkers represent the tumour burden and underlying genomic landscape of disease, and whether they can be used to shed light on mechanisms of therapeutic resistance in patients who may not have responded to specific therapies prior to death. In addition, collecting circulating biomarkers in the context of advanced metastatic disease where mutations are likely to be present at higher frequencies allows the optimisation of assays to detect genomic

Docusign Envelope ID: AB97AAF1-C9D6-4C1D-9E5C-84B38883CE23

aberrations so that more sensitive techniques can be developed for use in earlier stages of disease.

2.4. Intratumour heterogeneity and the tumour neo-antigen repertoire

The interplay between a tumour and the host immune system can help determine the immunogenicity of a tumour[42]. The process of immunosurveillance can select for subclones lacking immunogenic antigens[43] or subclones with reduced sensitivity to immune attack[44] allowing for clonal evolution and tumour progression; a process termed immunoediting[45]. Although evidence suggests that intratumour heterogeneity may limit the efficacy of conventional and targeted therapies, on account of factors such as sampling bias and clonal evolution[46], increased mutational diversity may lead to increased tumour neo-antigen production, and therefore potential new targets for immunotherapeutic drugs, such as immune checkpoint inhibitors[46-49]. Somatic tumour mutations resulting in mutant short peptide fragments (neo-antigens) are presented on the tumour cell surface by the major histocompatibility complex (MHC), promoting activation and expansion of tumour infiltrating lymphocytes (TILs). TILs, in particular CD8+ T-cells, have been shown to be associated with improved treatment response and prognosis in several tumour types[50], including breast[51], ovarian[52,53], melanoma[54] and lung[55]. The immunogenicity of a mutant peptide depends on its affinity for binding MHC class I ligands so that it can be presented to, and recognized by, CD8+ T cells[56]. The greater the extent of intratumour heterogeneity and mutational burden, the greater the repertoire of potentially exploitable neo-antigens within a tumour[57]. The collection of multi-region fresh tumour tissue at post-mortem can help determine the true impact of intratumour heterogeneity on the tumour neo-antigen repertoire, and how it can be used to predict immunotherapy response and therefore aid treatment stratification.

2.5. Cancer of unknown primary: identifying tumour origin and model of early metastasis

Cancer of unknown primary (CUP) represents between 1-5% of cancer diagnoses, where metastatic cancer is found but without a clear primary tumour. Most of these cases have a poor prognosis. In many, but not all cases, a primary cancer is eventually identified (or in some cases hypothesised). Post-mortem tissue analyses in cases of CUP have the potential to identify genetic aberrations and mutational signatures with distinct patterns, which may help identify the primary origin of tumours in patients who would otherwise have a poor prognosis with limited treatment options. In addition, post-mortem analyses and molecular tracing studies could track the patterns of metastatic spread in these tumours, as well as potentially identify drivers of metastasis[18]. Many cancers originally diagnosed as CUP demonstrate early metastasis, when the primary is small. Therefore, they are an ideal model system to study metastatic disease and potential drivers of the metastatic process. Future therapeutic strategies based on thorough understanding of how metastases spread and what drives the metastatic process would greatly change the outcome for CUP patients. Since metastatic disease is the most common cause of death in cancer patients, such strategies could potentially benefit a very large population of cancer patients.

2.6. Creating a digital archive for samples

A central digital archive of high quality microscopic scans of formalin fixed, paraffin embedded (FFPE) material matched to fresh tissue samples will be created across the study sites. It will be viewable and annotatable online by researchers across the study sites and other potential users. Histology images will be correlated with clinicopathological data and ultimately with genomic and proteomic data. The archive will facilitate selection of tissue samples for specific projects. The image files will also be of value as a substrate for study-wide morphology based studies, including *in silico* image analysis. Furthermore, the images can be correlated to paraffin blocks and used to guide tissue microarray (TMA) construction.

Docusign Envelope ID: AB97AAF1-C9D6-4C1D-9E5C-84B38883CE23

3. STUDY DESIGN

PEACE is a multi-centre prospective observational study intended to facilitate tissue donation from multiple tumour sites in the post-mortem setting. Potential study sites will be assessed to ensure that they have the infrastructure and ability to support specific programmes of research (such as TRACERx EVO). This has taken into account a likely attrition rate as a result of missed cases from consent to death. The PEACE protocol has been written in collaboration with oncologists, palliative care teams, molecular and histopathologists, ethicists, medical lawyers, and the patient advocate group 'Independent Cancer Patients' Voice' (ICPV). The study consent and tissue harvesting procedure have been developed in accordance with the Human Tissue Act 2004, and the consent allows for the use of collected samples in future ethically approved studies.

3.1. Study Objectives

Access to the resource of tissue and blood samples facilitated by the study will enable cancer research across different disciplines with the intention of developing a greater understanding of basic tumour biology, such as genetic and phenotypic mechanisms involved in tumourigenesis and the metastatic process. Other future research aims in the different tumour types may include:

- To define the extent of intratumour heterogeneity
- To define the immunological landscape and the tumour neo-antigenic repertoire
- To decipher polygenic and convergent mechanisms of resistance to targeted therapies by linking with national programmes, such as the TRACERx EVO
- To explore the relationship of CTCs and cfDNA with tumour burden in metastatic cancers, and the degree to which such circulating biomarkers are representative of the underlying tumour landscape
- To decipher the origins and evolution of metastatic subclones through integration with primary tumour longitudinal studies, such as TRACERx EVO
- To elucidate and explain the breadth of tumour cell histological appearances as signifiers of cell biology linked to genomic/proteomic features of tumour clones
- To develop in vivo models, such as tumour organoid models that efficiently represent the genetic diversity of late stage disease
- To identify mutational aberrations associated with prior treatment with radiotherapy
- To investigate the somatic mutational burden and evolution of normal and diseased tissue
- To investigate the epigenetic profile/methylome of different cancers

3.2. Study Activation

UCL CTC will ensure that all Study documentation has been reviewed and approved by all relevant bodies and that the following have been obtained prior to activating the Study:

- Research Ethics Committee approval
- 'Adoption' into NIHR portfolio
- NHS Permission

Docusign Envelope ID: AB97AAF1-C9D6-4C1D-9E5C-84B38883CE23

- Adequate funding for central coordination
- Confirmation of sponsorship
- Adequate insurance provision

Docusign Envelope ID: AB97AAF1-C9D6-4C1D-9E5C-84B38883CE23

4. SELECTION OF SITES/SITE INVESTIGATORS

4.1. Site Selection

In this protocol study 'site' refers to a hospital where study-related activities are conducted.

Sites must be able to comply with:

- The ability to perform tissue harvests in the post-mortem setting as well as processing and storage requirements
- Blood sample collection at baseline and subsequent follow up visits and all requirements of the study protocol
- Data collection requirements using an electronic data capture system, including adherence to eCRF guidance and submission of source document timelines as per section 9
- Necessary infrastructure to support this study

4.1.1. Selection of Principal Investigator and other investigators at sites

Sites must have an appropriate Principal Investigator (PI), i.e. a health care professional authorised by the site to lead and coordinate the work of the study on behalf of the site. Other investigators at site wishing to participate in the study must be trained and approved by the PI. All investigators must be either clinicians, including palliative care doctors, or pathologists.

4.1.2. Training requirements for site staff

All site staff must be appropriately qualified by education, training and experience to perform the study related duties allocated to them, which must be recorded on the site delegation log.

CVs for all staff must be kept up-to-date, signed and dated and copies held in the Investigator Site File (ISF). An up-to-date, signed copy of the CV for the PI must be forwarded to UCL CTC upon request.

4.2. Site initiation and Activation

4.2.1. Site initiation

Before a site is activated, the UCL CTC study team will perform a site initiation by either on-site visit/telephone/ videoconference which the PI, the tissue collector/research nurse and lab technician must attend (as well as other members of the research team at site as appropriate). The site will be trained in the management of the study and essential documentation required for the study will be checked.

Docusign Envelope ID: AB97AAF1-C9D6-4C1D-9E5C-84B38883CE23

4.2.2. Required documentation

The following documentation must be submitted by the site to UCL CTC prior to a site being activated by the UCL CTC Study team:

- Study specific Site Registration Form (identifying relevant local staff)
- All relevant institutional approvals/confirmation of capacity and capability (e.g. local NHS permission/confirmation of capability and capacity)
- A completed site delegation log that is initialled and dated by the PI (with all tasks and responsibilities delegated appropriately)
- Completed site contacts form (with contact information for all members of local staff)
- A copy of the PI's current CV that is signed and dated (with documented up to date GCP training, or copy of GCP training certificate)

In addition, the following agreements must be in place:

- a signed Clinical Trial Site Agreement (CTSA) between the Sponsor and the relevant institution (usually an NHS Trust/Health Board)

4.2.3. Site activation letter

Once the UCL CTC Study team has received all required documentation and the site has been initiated, a site activation letter will be issued to the PI, at which point the site may start to approach patients.

Following site activation, the PI is responsible for ensuring:

- adherence to the most recent version of the protocol;
- all relevant site staff are trained in the protocol requirements;
- appropriate recruitment and follow-up of patients in the study;
- timely completion and submission of eCRFs;

Docusign Envelope ID: AB97AAF1-C9D6-4C1D-9E5C-84B38883CE23

5. INFORMED CONSENT

Written informed consent from the patient, a nominated representative or a person in a qualifying relationship with the patient (if a nominated representative has not been appointed) will take place as a prerequisite for study entry (i.e. consent for post-mortem tissue collection in accordance with the Human Tissue Act 2004, the acquisition of tissue samples from sites of disease and normal tissue, blood sample collection for research purposes, and for retrospective demographic and clinicopathological data collection as well as archival sample collection).

After death, consent can be obtained from a nominated representative or a person in a qualifying relationship. The nominated representative needs to have been appointed by the patient when they were alive. The qualifying relationship needs to have existed when the patient was alive.

If the consent process cannot be undertaken in person, consent can be obtained over telephone. Relevant PIS and consent forms should be posted in good time before the telephone consent conversation is due to take place. Once consent form is signed by the patient, a nominated representative or a person in a qualifying relationship with the patient after death (if a nominated representative has not been appointed) and a witness, forms should be posted and returned to the site team. The site team member who obtained consent will then countersign the consent form with the date telephone conversation took place and the date the consent form was received.

Consenting procedures will conform to GCP and local and national regulations. It will be strongly encouraged that patients discuss participation in the study with their family, relatives and next of kin.

In line with local trust policy, consent of patients, follow-up visits and study sample collection may occur at a patient’s home, if appropriate. Telephone follow up assessments can also be performed, if appropriate.

Sites are responsible for assessing a patient’s capacity to give informed consent.

Please note that the study is unfortunately unable to cover or contribute towards funeral expenses for patients who are recruited to PEACE.

The PI, or, where delegated by the PI, other appropriately trained site staff, are required to provide a full explanation of the study and all relevant procedures to each patient, nominated representative or person in a qualifying relationship prior to study entry. During these discussions, the current approved relevant information sheet for the study should be discussed with the appropriate person giving consent.

Patients should be given adequate time to consider and discuss participation in the study or their nominated representative/person in a qualifying relationship on their behalf. If appropriate, consent can be provided on the same day, either by the patient or after death by their nominated representative or the person in a qualifying relationship. Written informed consent on the current approved version of the consent form for the study must be obtained before any study-specific procedures are conducted. The discussion and consent process must be documented in the patient notes.

Site staff are responsible for:

- checking that the correct (current approved) version(s) of the patient information sheet(s) and consent form(s) are used;
- checking that information on the consent form(s) is/are complete and legible;
- checking that the patient/nominated representative/person in a qualifying relationship has completed/initialed all relevant sections and signed and dated the form;
- checking that an appropriate member of staff has countersigned and dated the consent form(s) to confirm that they provided information to the patient, their nominated representative or the person in a qualifying relationship;
- checking that a witness has countersigned and dated the consent form, if this is a requirement by the mortuary performing the tissue harvest (if tissue harvest could be performed at UCLH, witness signature is required);
- checking that an appropriate member of staff has made dated entries in the patient's medical notes relating to the informed consent process (i.e. information given, consent signed etc.);
- adding details of all consented patients to the registration form and database (See section 11.2 for further details);
- following registration: adding the patient study number to all copies of the consent form(s), which should be filed in the patient's medical notes and investigator site file;
- giving the patient or their nominated representative/person in a qualifying relationship a copy of the signed consent form and information sheet.

The right of the patient to refuse to participate in the study without giving reasons must be respected. All patients are free to withdraw at any time. Also refer to section 15 (Withdrawal of Patients).

At the time of consent, a full explanation of the tissue harvesting procedure along with discussion and adequate time for reflection will be given to the patient or their nominated representative/person in a qualifying relationship. Individuals and relatives will be able to discuss this process fully and ask any questions. Staff seeking consent for tissue harvesting after death will be trained in how to obtain valid consent.

5.1.1. Consent given by the patient prior to death

If consent is taken from the patient they will be approached in the outpatient, community, and hospital settings for study recruitment and consent. Patients will be given a patient information sheet and asked to sign the consent form if and when they choose to enter the study.

Sites must ensure that all patients have been given the current approved version of the patient information sheet and are fully informed about the study. Patients, where applicable, must have confirmed their willingness to take part in the study by signing the current approved consent form.

Sites must assess a patient's ability to understand verbal and written information in English and whether or not an interpreter would be required to ensure fully informed

Docusign Envelope ID: AB97AAF1-C9D6-4C1D-9E5C-84B38883CE23

consent. If a patient requires an interpreter and none is available, the patient should not be considered for the Study.

5.1.2. Consent given by an individual other than the patient after the patient's death (applies only to England, Wales and Northern Ireland)

In the event where it has not been possible to obtain consent from a patient prior to death, consent may be sought from either their 'nominated representative', or where they had not appointed a nominated representative, someone who was in a 'qualifying relationship' with the patient immediately before they died. The principles of the HTA Code of Practice on Consent
(https://www.hta.gov.uk/sites/default/files/HTA%20Code%20A_1.pdf , paragraphs 86-9

The list of qualifying relationships is detailed on page 9 of this Code of Practice, as shown below:

1. Spouse or partner (including civil or same sex partner) The HT Act states that, for these purposes, a person is another person's partner if the two of them (whether of different sexes or the same sex) live as partners in an enduring family relationship.
2. Parent or child >18yrs (in this context a child may be a biological or adopted child)
3. Brother or sister
4. Grandparent or grandchild
5. Niece or nephew
6. Stepfather or stepmother
7. Half-brother or half-sister
8. Friend of long standing.

Consent is needed from only one person in the hierarchy of qualifying relationships and should be obtained from the person ranked highest. If a person high up in the list refuses to give consent, it is not possible to act on consent from someone further down the list. If there is no one available in a qualifying relationship to make a decision on consent, it is not lawful to proceed with removal, storage or use of the deceased person's body or tissue for scheduled purposes.

If after death those close to the patient object to the study, for whatever purpose, when the patient (or their representative) has explicitly consented, the study team should seek to discuss the matter sensitively with them. Emphasis in these difficult situations should be placed on having an open and sensitive discussion with those close to the deceased where the process is explained fully to them. The study team should also consider the impact of going ahead with a procedure in light of strong opposition from the family, despite the legal basis for doing so. For example, the study team may consider that carrying out an anatomical examination would leave relatives or family members traumatised (or lead to their objections), despite the patient having consented to this whilst alive. In such circumstances, a study tissue harvest in a post-mortem setting should not go ahead.

Docusign Envelope ID: AB97AAF1-C9D6-4C1D-9E5C-84B38883CE23

Sites must ensure that a nominated representative or a person in a qualifying relationship with the patient have been given the current approved version of the nominated representative/ person in a qualifying relationship information sheet and are fully informed about the study.

Docusign Envelope ID: AB97AAF1-C9D6-4C1D-9E5C-84B38883CE23

6. SELECTION OF PATIENTS

All patients who fulfill the inclusion criteria will be eligible for the study. Patients will, mostly, be those already part of longitudinal studies, such as TRACERx EVO. General practitioners will be notified of patient study entry by post.

The aim of the study is to perform tissue harvests to enable sample collection and in-depth genomics analyses with high-quality clinical data collection, creating an unprecedented tissue and blood resource. It is anticipated that a process of selection may occur in each centre where the interests of clinicians and scientists, and the existing national clinical trials and longitudinal studies with which PEACE can link into in order to enable longitudinal sampling, will influence the patients that are recruited. Involvement of palliative care team will be encouraged and supported at each site to help with patient recruitment and consent. The protocol allows for the retrieval of bodies from hospices.

Pre-registration Evaluation

Patients or their representative must give written informed consent **before** any study specific procedures may be carried out. The following assessments or procedures are required to evaluate the suitability of patients for the study:

- histological or cytological confirmation of solid malignancy with metastatic disease (where the site of origin is known or unknown) or;
- if confirmed diagnosis of a primary brain tumour, this may be with or without local or distant metastatic disease
- confirmation that there is no history of intravenous drug abuse within the last 5 years
- confirmation of diagnosis for high-risk infections (e.g. HIV/AIDS, hepatitis B/C, tuberculosis and Creutzfeldt-Jacob disease) unless patient case is of a particular scientific interest and agreed in advance with local mortuary staff and pathologist.

6.1. Screening Log

A screening log must be maintained by the site and kept in the Investigator Site File. This must record each patient identified with a relevant cancer and the reasons why they were not registered in the study if this is the case. The log must be sent to UCL CTC when requested, with patient identifiers removed prior to sending where applicable.

6.2. Patient Eligibility

Study team and CI should ensure consideration is given to inclusion of all populations, e.g. see ASCO guidance on 'broadening eligibility criteria to make clinical trials more representative' at:

https://www.asco.org/research-data/clinical-trials/clinical-trial-eligibility-criteria
https://ascopubs.org/doi/full/10.1200/JCO.2017.73.7916.

To maximise generalisability of results, trial enrolment criteria should strive for inclusiveness. The rationale for excluding particular patients/patient groups should be clearly explained and reflect expected toxicities associated with the therapy under

Docusign Envelope ID: AB97AAF1-C9D6-4C1D-9E5C-84B38883CE23

investigation (if written justification is not provided in the protocol retain written comments in TMF).

There will be no exception to the eligibility requirements at the time of registration. Ensuring patient eligibility is the responsibility of the PI or other delegated Investigator(s). Queries in relation to the eligibility criteria must be addressed prior to registering a patient. Patients are eligible for the study if all the inclusion criteria are met and none of the exclusion criteria applies. CI must review criteria carefully to ensure they are appropriate for intended purpose.

6.2.1. Inclusion criteria

- Age 18 years or older
- Confirmed diagnosis of any form of solid malignancy with metastatic disease (where the site of origin is known or unknown), with the exception of primary brain tumour in which there may not be evidence of metastatic disease
- Oral and written informed consent from patient to enter the study and to undergo tissue harvesting after death or informed consent from a nominated representative or a person in a qualifying relationship after the patient has died.

6.2.2. Exclusion criteria

- Medical or psychiatric condition that would preclude informed consent
- History of intravenous drug abuse within the last 5 years
- Confirmed diagnosis for high-risk infections (e.g. HIV/AIDS-positive, hepatitis B/C, tuberculosis and Creutzfeldt-Jacob disease) unless patient case is of a particular scientific interest and agreed in advance with local mortuary staff and pathologist.

6.2.3. Recruitment Selection

Patients recruited into the study will mostly be part of a longitudinal study, such as TRACERx EVO, or fulfil the criteria within an existing approved research proposal, or a proposal that is soon to be submitted for approval.

Any queries relating to a proposal or whether a patient fulfils proposal must be raised with UCL CTC (ctc.peace@ucl.ac.uk)

- In exceptional circumstances, for example cases of particular interest, recruitment may be outside of a research proposal provided this has been agreed with the UCL CTC.

Docusign Envelope ID: AB97AAF1-C9D6-4C1D-9E5C-84B38883CE23

7. REGISTRATION PROCEDURES

7.1. Registration

Patient registration will be performed via a remote electronic data capture system hosted by UCL CTC. Following pre-registration evaluations (as detailed in section 6.1), confirmation of eligibility and consent of a patient at a site, the registration should be completed on OpenClinica:

https://ucl.openclinica.io/

A study number will be assigned for the patient once the registration process has been completed and must be recorded by the site on the master subject log. If there are any difficulties, sites should contact the UCL CTC:

Telephone number:	+44 (0)20 7679 9016
UCL CTC Office hours:	09:00 to 17:00 Monday to Friday, excluding Bank Holidays

Once a patient has been registered onto the study they must be provided with the following (or their nominated representative or a person in a qualifying relationship must be provided with):

- A copy of their signed consent form and patient information sheet
- A patient contact card with details of the study team and who to call in the event of death or hospital admission for progression. Patients advised to carry this with them at all times while participating in the study

UCL CTC will confirm patient registration and add the patient to the PEACE sample tracker database to enable the tracking of study samples.

After registration into the study, the patient's general practitioner (GP) should be informed of the patient's involvement in the study by the site completing and sending the completed GP letter.

Docusign Envelope ID: AB97AAF1-C9D6-4C1D-9E5C-84B38883CE23

8. STUDY SPECIFIC PROCEDURES

8.1. Blood Sample Collection

Patients entered into the study will be asked to give consent for blood sampling at baseline and subsequent time points whilst alive and blood and tissue collection after death. In line with local trust policy, consent of patients, follow-up visits and study sample collection may occur at a patient's home, if appropriate.

The blood samples collected will be used for the purpose of germline DNA extraction, cfDNA, CTC and immunological analyses. Samples will be processed according to the Trial Specific Procedures for consistency across all recruiting sites. In some cases, samples may be processed by collaborators at other sites, in particular if improved methods of processing and analysis exist in collaborating laboratories. Blood samples will be tracked at each site using the PEACE sample tracker database.

The following blood samples will be collected at baseline:

- 10ml for germline DNA
- 20ml for plasma and subsequent cfDNA and cytokine and metabolite analyses
- 20ml (2x special tube) for CTCs (only required for selected patients, as confirmed by CTC)
- 40ml for immunological analyses (only required for patients who are also participating in TRACERx EVO and selected lung patients)
- 3ml (special tube) for T-cell and B-cell receptor sequencing

If possible, the following blood samples will be collected at subsequent follow-up visits after the baseline samples:

- 20ml for plasma and subsequent cfDNA and cytokine and metabolite analyses
- 20ml (2x special tube) for CTCs (only required for selected patients, as confirmed by CTC)
- 40ml for immunological analyses (only required for patients who are also participating in TRACERx EVO and selected lung patients)
- 3ml for (special tube) T-cell and B-cell receptor sequencing
- 10ml Germline (only if not previously collected at baseline visit)

Ideally these samples will be collected at the time of subsequent disease progression and/or at the same time as imaging is performed.

If possible, up to 93ml (refer to baseline sampling for breakdown) of blood may also be taken at the time of the tissue sampling after death:

- 10ml for germline DNA
- 20ml for plasma and subsequent cfDNA and cytokine and metabolite analyses

Docusign Envelope ID: AB97AAF1-C9D6-4C1D-9E5C-84B38883CE23

- 20ml (2x special tube) for CTCs (only required for selected patients, as confirmed by CTC)
- 40ml for immunological analyses (only required for patients who are also participating in TRACERx EVO and selected lung patients)
- 3ml (special tube) for T-cell and B-cell receptor sequencing

Refer to TSP 1 – Blood Sample Processing for further details.

8.2. Imaging Prior to Tissue Sampling

Imaging performed prior to death will help in sampling of widespread disease, to aid the pathologist in the accurate sampling of tumour tissue and will be of particular value in the detection of occult intracranial and bone metastasis. In addition, the images will facilitate quantitative mapping and measures of anatomical patterns of metastasis and whole body tumour burden.

For each patient, a copy of the scan(s) images and associated report(s) may be obtained; ideally this will be in real-time, however scans will also be accepted retrospectively. Scan images will be uploaded via Smart Portal database, sent via PACS link or physically in the form of a CD. Scan reports will be uploaded via the OpenClinica database. Scan images and associated reports performed since the patient's diagnosis may be requested as well as the imaging that was used to guide the tissue sampling at the tissue harvest (if applicable).

Scans must be pseudo-anonymised and labelled with the patient's PEACE Study number and date of scan (e.g. PEA001_dd/mmm/yyyy) in the file name – where month is written in letters (e.g. APR for April).

8.3. Biological Tissue Samples

Tissue sampling will be performed in the mortuary of a recruiting or affiliated hospital provided a license exists to perform post-mortem procedures, by a pathologist in accordance with the Human Tissue Act 2004.

If the patient does not die at a PEACE participating hospital, or dies at home or in a hospice , the body will be transferred to a PEACE participating hospital, in order to perform the tissue sampling.

If a patient entered into the study is admitted to hospital, contact will be made with the medical ward team to explain the study and the potential outcome within the study after death. The study team will encourage patients to pass on the contact details of the study team to whomever they wish, so that study staff may also be notified by someone outside of the medical team with regards to the patient's admission to hospital and subsequent death. In case this does not occur, hospital admissions may be tracked on a daily basis to confirm admission.

Whether the patient dies in hospital, at home, or in a hospice, a copy of the consent forms will be shown to the mortuary technician and pathologist performing the tissue sampling,. The staff viewing the consent form are NHS staff who are bound by the NHS Confidentiality Code of Practice and NHS Information Governance. Any additional checks required as per local policy, should also be conducted (e.g correlation of the Medical Certificate of Cause of Death (MCCD)/death certificate with the consent forms). The consent form should also be checked to verify if the patient wished to limit the sampling of tissue during tissue harvest.

If a coroner's post-mortem is indicated for a patient entered into the study, the study team will consider tissue sampling alongside this process. This will be asked for separately in the study consent form.

Tissue harvests which are known to carry a higher infectious risk, as outlined in the RCPath guidelines, are not excluded from the study, although these should be limited to individual cases of particular scientific interest. In these cases the local mortuary staff and pathologists must be informed of the high-risk nature of the case in advance and be willing to perform the tissue harvests in line with their departmental high risk autopsy SOPs. In addition, it is recommended that the mortuary consider utilising the following

1. A Human Tissue Authority accredited mortuary autopsy facility with Hazard Group 3 pathogen infection prevention facilities, preferably with an Airborne Infection Isolation Room (AIIR), and instruments
2. Scrubs and appropriate HG3 PPE for pathologist, APT and research assistant

It is strongly advised that the tissue collection for these specific cases is taken on entirely by the pathologists and mortuary staff rather than the full PEACE tissue collection team. This is to limit those present within the PM room to a minimum number of staff and specifically to those with experience of high-risk autopsies.

The aim of the study is to obtain high quality tissue samples, which is more likely to be the case the sooner after death the tissue sampling is performed, ideally within 48 hours after death, but preferably within 24 hours. Earlier tissue harvests in a post-mortem setting with shorter ischaemic time are likely to yield better DNA, RNA and protein quality. In a study by van der Linden and colleagues, the quality of RNA in post-mortem samples has been previously assessed (58). Hypoxia, body mass index (BMI), hypertension and dyslipidaemia were not found to influence RNA integrity (measure by the RIN score), but fever prior to death and diabetes did have an adverse effect on RNA integrity. As expected, the RNA integrity decreased with increasing time after death. Samples obtained using minimally invasive autopsy methods, such as CT-guided biopsies, were less degraded compared to those obtained using conventional autopsy methods, probably due to shorter ischaemic times. RNA quality for gene expression analyses was reasonable >15 hours after death.

Biological samples retained for the study will be labeled using barcoded labels. In addition, all samples will be labelled with the PEACE study number and other patient identifiers (such as name and date of birth) will be removed prior to storage in order to maintain patient confidentiality. Researchers will therefore not have access to any details that identify the patient and will see all data in pseudo-anonymised form in almost all circumstances.

Docusign Envelope ID: AB97AAF1-C9D6-4C1D-9E5C-84B38883CE23

Patients or their representative(s) will be asked to give written informed consent for the collection of blood, tissue samples after death from both primary and metastatic tumours, as well as normal and uninvolved tissue. In addition, consent will be requested to obtain bone marrow samples and to remove the prostate and/or brain. Consent will include retention of material for future ethically approved research as well as the photography of samples taken at the time of collection. In some cases samples may be processed by collaborators, in particular if improved methods of processing and analysis exist.

Samples may be stored at each recruiting site until the end of the study at which point either an extension of the study ethics approval will be sought. Or the samples may be transferred to a site with a storage license for post-mortem tissue. Samples will be tracked using a barcode scanner and the PEACE sample tracker database created by the Cancer Research UK & UCL Cancer Trials Centre.

Tissue will be collected comprehensively from multiple sites of tumour and normal tissue, guided by imaging, findings at tissue harvest or high clinical suspicion. Sampling will include all tumour tissue (this may involve removal of organs to allow sampling) including samples of morphologically distinct regions within those tumours to assess intratumour heterogeneity. Sampling will also include extensive background normal tissue sampling of diseased organs, in the case of prostate and brain cancer this may involve sampling of the entire prostate/brain. Any remaining tissue will be returned to the body. These tumours will be photographed to ensure that samples and regions can be orientated within the body and are mapped accordingly. All data files, including photographs, will be stored on a password-protected server shielded by firewalls. Samples from normal adjacent tissue will also be collected for comparison. Blood samples will be collected once the patient enters the study for germline DNA, cfDNA, CTCs analyses, immunological analyses and T-Cell Receptor Sequencing. Patients or their representative will also be asked to give consent for access to archival samples, which may have been collected in different hospitals, for research purposes. If present at the time of sample collection after death, the following samples may also be collected: cerebrospinal fluid, pleural fluid, pericardial fluid, peritoneal fluid and urine.

Where indicated, smaller scale sampling of normal tissues from involved sites may take place. For example, in patients treated with immunotherapeutic drugs, such as checkpoint inhibitors, where it is of research interest to establish rates of subclinical drug toxicity in relation to clinical symptoms captured by clinical data collection. In addition, bone marrow samples may be obtained.

Tissue samples will be collected in the form of fresh, snap frozen and FFPE tissue. Samples will be processed and stored according to the Trial Specific Procedures (TSPs). Samples requiring the expertise of other collaborating laboratories may be transferred to these laboratories for immediate processing and storage according to the study TSPs. For example, the processing and biobanking of blood for CTCs and fresh tissue and blood for immunological analyses. All patients or their representatives will be asked to give consent for samples to be used within the PEACE study and in future ethically approved research.

Samples will be labelled with a barcode and the PEACE study number so that researchers will not have access to details that identify the patient and will see all data in pseudo-anonymised form. Key study individuals will be able to link the PEACE study number with the patient identifiers to ensure that on receipt the samples have been associated with the correct study, and to enable the accurate collection of clinicopathological and demographic data.

Refer to TSP 2 – Tissue Sample Processing for further details.

8.4. Planned Sample Analysis

The aim of tissue and blood sample analysis is to identify changes correlating with retrospective tumour behaviour and treatment response referring to the demographic and clinicopathological data collected. Collection of normal tissue from involved and uninvolved organs may be used to assess the tumour microenvironment and background changes related to certain types of treatment, such as immune checkpoint inhibitor therapies. Samples obtained from the bone marrow may be used to determine the presence of micrometastatic disease and to define the nature of such cellular populations. The prostate/brain may be removed in their entirety to allow detailed spatial sampling. A number of techniques may be employed in the analysis. Analysis from PEACE may be combined with analysis performed on data from other trials/studies. Results from the analysis will not be fed back to patient relatives or friends since these are experimental analyses. The list below is not exhaustive, and analytical techniques may be employed depending on initial results, new data, and the potential future development of new techniques.

Potential analyses include:

- Sequence analysis of genes known to be mutated in the specific type of cancer and their relationship to histopathological parameters, treatment history, sites of metastatic disease, tumour behaviours and clinical outcome
- RNA analysis from blood, including T cell receptor sequencing
- Characterisation of immune subset, diversity, function and transcriptional profile and their relation with cancer cachexia.
- Analysis of genomic instability status (chromosomal instability) using flow cytometry and/or image cytometry
- High-throughput mRNA and miRNA/non-coding RNA expression profiling using RNAseq or mRNA microarray expression platforms to detect alterations associated with specific histopathological and molecular subtypes of the specific cancer
- Quantitative real time polymerase chain reaction (RT-qPCR), and reverse transcription PCR to confirm alterations in genes and gene expression.
- RNA sequencing of bulk and sorted tumour, stroma and tumour infiltrating immune subsets including T-cell and B-cell sequencing, may also be performed on tissue samples (normal and cancerous)
- Next generation sequencing analysis of cancer genomes (both DNA and RNA-seq) with DNA copy number/SNP-CGH analysis of germline and tumour DNA
- Next generation sequencing, proteomic and methylome analysis of single cells derived from tumour and normal tissue, CTCs from blood samples and disseminated tumour cells (DTCs) from bone marrow samples, to allow the

Docusign Envelope ID: AB97AAF1-C9D6-4C1D-9E5C-84B38883CE23

recreation of a 4D lineage map of cancer development.

- Next generation sequencing analysis of cancer genomes and epigenomes (both DNA, RNA-seq and methylation analyses) with DNA copy number/SNP-CGH analysis of germline and tumour DNA
- Phosphoproteomic analyses to investigate signalling pathway aberrations in advanced malignancy.
- Immunohistochemistry (IHC) and proteomic analysis to detect loss or gain of protein expression
- Morphological microscopic/ultramicroscopic studies linking genomic/proteomic changes to tumour cell biology
- Immune analysis: profiling of tumour infiltrating lymphocytes using IHC and multi-colour flow cytometry for each sample obtained from different regions within a tumour
- Further validation of the alterations in genes and gene expression by using chromosomal in situ hybridisation (CISH) and fluorescent in situ hybridization (FISH).
- Construction of Tissue Microarrays for the evaluation of molecular markers, allowing rapid analysis in tumour samples using immunohistochemistry and the determination of the significance of each marker with regard to different variables, such as response to treatment and survival
- Expression and/or sequence analysis of genes associated with cytotoxic sensitivity or cancer progression
- Protein expression and sequence analysis in germline and tumour DNA
- CTCs will be isolated from whole blood using CellSearch™ (Jannsen Diagnostics) or other available novel technologies
- cfDNA will be isolated from plasma according to standard protocols. Molecular analyses will mirror those described above
- Plasma analyses for cytokine and metabolites
- Establishing cell lines from tissue collected after death
- Establishing *in vivo* models from tissue collected after death
- Blood platelets mRNA, IncrRNA and microRNA expression analyses by NGS or RT-PCR methods
- DNA analysis in blood platelets by NGS or RT-PCR methods
- Analysis of normal tissue where appropriate
- Samples of tumour and normal tissue will be used for the establishment of patient-derived organoids to generate pre-clinical models that can be used for functional in vitro experiments at the level of an individual patient. This will provide the opportunity to generate and test hypotheses that directly link to other data sources, e.g. DNA sequencing or PDX models.
- Single cell sequencing of Tumour infiltrating lymphocytes and peripheral blood mononuclear cells. DNAseq and TCRSeq
- Analysis of extrachromosomal DNA in lung TRACERx/PEACE patients
- Analysis of the microbiome and the non-human metagenome in normal and human tissues utilising techniques including, but not limited to, whole genome sequencing and 16s RNA sequencing.

- Viable patient-derived fibroblasts may be used to make induced pluripotent stem cells. The cells will be characterised and maintained as per International Society for Stem Cell Research Guidelines for Stem Cell Research and Clinical Translation. The cells will be stored in the central cell services facilities at the Francis Crick Institute and the University College London. The distribution of the cells will be strictly controlled by the PEACE consortium in guidance from the local ethics committee. These cells would be differentiated into cell types found in normal lung and lung cancer microenvironment depending on the need of the relevant research project. The cells may be genetically modified as per the experimental requirement, but gametes would not be produced neither the cells will be used for cell therapy purposes.

Sites must submit a proposal to the Study Governance Board (section 10.5.2) if they wish to access samples or data collected within PEACE (to utilise samples collected locally and/or nationally).

8.5. Creating a Central Digital Archive

Each site will be asked to send an H&E slide corresponding to the collected tissue samples after death to UCL Cancer Institute for scanning. These slides will be returned to sites subsequently.

These digital H&E images from regions matched to frozen tissues will be collected and organised into a web-enabled database with in-browser image viewing, enabling users to directly examine archived tissues. This will facilitate tissue selection for studies, but will also be invaluable as a substrate for single or multi-user targeted histopathological annotation, to guide core selection for TMA construction, for direct manual or automated morphological studies, and possible unsupervised/semi-supervised image analytical methods.

8.6. Collection of Clinicopathological and Demographic Data

Detailed clinical data will be collected for each patient, including patient medical and treatment history, using electronic case report forms (eCRFs). Demographic data, including full UK home postcode(s) of the patient for the 5 years prior to their cancer diagnosis, will be collected. This data may be collected from hospital notes for deceased patients. This will be a password-protected server shielded by firewalls using the patient's study number.

Docusign Envelope ID: AB97AAF1-C9D6-4C1D-9E5C-84B38883CE23

9. DATA MANAGEMENT AND DATA HANDLING GUIDELINES

Data will be collected from sites using an electronic data capture system.

Source data are contained in source documents and must be accurately transcribed on to the electronic data capture system. Examples of source documents are hospital records which include laboratory and other clinical reports etc.

Where copies of supporting source documentation (e.g. autopsy reports, pathology reports, CT scan images/reports etc.) are being submitted to UCL CTC, the patient's study number must be clearly indicated on all material and any patient identifiers completely removed prior to sending to maintain confidentiality.

Please note that, for this trial, patients/representative must consent to their NHS/CHI number being supplied to UCL CTC.

9.1. Data Entry

All entries made to the electronic data capture system must be by staff who are listed on the site staff delegation log and authorised by the PI to perform this duty. The PI is responsible for the accuracy of all data reported in the electronic data capture system.

9.2. Missing Data

To avoid the need for unnecessary data queries, data entered must be checked at site to ensure there are no blank fields. When data are unavailable, the status of the field should be changed to 'Not Available'. When data are unknown and there is the option in the dropdown fields, please enter the value "NK" (only use if every effort has been made to obtain the data).

9.3. Timelines for Data Entry

Data entry to the electronic data capture system must be completed as soon as possible after patient visit and within 1 month of the patient being seen.

9.4. Data Queries

Data entered into the electronic data capture system will be checked for completeness, accuracy and consistency, including checks for missing or unusual values. Queries and guidance for resolution will be raised directly on the electronic data capture system and/or sent to the data contact at site.

Docusign Envelope ID: AB97AAF1-C9D6-4C1D-9E5C-84B38883CE23

10. INCIDENT REPORTING, STUDY MONITORING AND OVERSIGHT

10.1. Incident Reporting

Organisations must notify UCL CTC of all deviations from the protocol or GCP immediately. UCL CTC may require a report on the incident(s) and a form will be provided if the organisation does not have an appropriate document (e.g. Trust Incident Form).

If site staff are unsure whether a certain occurrence constitutes a deviation from the protocol or GCP, the UCL CTC study team can be contacted immediately to discuss.

10.2. On-Site Monitoring

The degree of on-site monitoring will be proportionate to the objective, purpose, phase, design, size, complexity, endpoints and risks associated with the trial.

Details of monitoring activities will be included in the trial monitoring plan and conveyed to sites during initiation which will be provided to Sites. The trial monitoring plan will be kept under review during the trial and updated information provided to sites as necessary.

Sites will be sent a letter in advance of any on-site monitoring visits, confirming when a visit is scheduled to take place. The letter will include a list of the documents to be reviewed, interviews that will be conducted, planned inspections of the facilities and who will be performing the visit.

Monitoring Follow Up

Following a monitoring visit, the Trial Monitor/Trial Manager will provide a follow up email to the site, which will summarise the documents reviewed and a statement of findings, incidents, deficiencies, conclusions, actions taken and/or actions required. The PI at each site will be responsible for ensuring that monitoring findings are addressed in a timely manner, and by the deadline specified.

10.3. Centralised Monitoring

UCL CTC or its representatives perform centralised monitoring and requires the submission of the following documents by sites to UCL CTC for review: staff delegation logs, screening logs, updated GCP and CV (if applicable at the time) and ISF checklists form completeness. Additional documents may also be requested for the purposes of remote source data review when it is not possible to check these on-site. Expectations for document submission will be explained during site initiation and UCL CTC or its representatives will send emails to sites requesting the documents when required. Additional documents may also be requested for the purposes of remote source data verification/review when it is not possible to check these on-site'.

Docusign Envelope ID: AB97AAF1-C9D6-4C1D-9E5C-84B38883CE23

Sites will be requested to conduct quality control checks of documentation held within the Investigator Site File at the frequency detailed in the study monitoring plan. Checklists detailing the current version/date of version controlled documents will be provided for this purpose.

10.4. 'Triggered' On-Site Monitoring

Additional on-site monitoring visits may be scheduled following UCL CTC review and/or where there is evidence or suspicion of non-compliance at a site with important aspect(s) of the study protocol/GCP requirements. Sites will be sent a letter in advance outlining the reason(s) for the visit and confirming when it will take place. The letter will include a list of the documents that are to be reviewed, interviews that will be conducted, planned inspections of the facilities and who will be performing the visit.

Following a monitoring visit, the study coordinator will provide a follow up email to the site, which will summarise the documents reviewed and a statement of findings, incidents, deficiencies, conclusions, actions taken and/or actions required. The PI at each site will be responsible for ensuring that monitoring findings are addressed in a timely manner, and by the deadline specified.

UCL CTC or its representatives will assess whether it is appropriate for the site to continue participation in the Study.

10.5. Oversight Committees

10.5.1. PEACE Consortium

The consortium will include the Chief Investigator, clinicians and experts from relevant specialities and PEACE study staff from UCL CTC. Meetings will be held at appropriate intervals to discuss the study. The group will send updates to PIs (via newsletters or at Investigator meetings) and to the NCRI Lung Clinical Studies Group.

All PIs will be kept informed of substantial amendments through their nominated responsible individuals.

10.5.2. Study Governance Board (SGB)

A PEACE Study Governance Board was established and consists of clinical and/or scientific leads representing all or specific tumour types and at least three external members, such as representatives from Cancer Research UK, Cancer Research Technology and Patient and Public Involvement groups. Each member will sign a charter provided by UCL CTC. The SGB will meet when required and continue to oversee research conducted under approved PEACE research proposals prior to October 2024 and resulting publications.

A PEACE Study Oversight Group (SOG) will include the Chief Investigator, clinicians and experts from relevant longitudinal studies (e.g. TRACERx EVO). The SOG will meet when

Docusign Envelope ID: AB97AAF1-C9D6-4C1D-9E5C-84B38883CE23

required and oversee study activities for patients recruited post October 2024, use of any samples collected throughout the duration of the study and all publications.

All PIs will be kept informed of substantial amendments through their nominated responsible individuals.

Study sites will be asked to review the following at necessary intervals and submit a report to the SGB meeting;

- Study set up and relevant institutional approvals/confirmation of capacity and capability
- Protocol amendment (depending on nature of amendment)
- Patient recruitment and consent
- Number of tissue harvests per site
- Number of tissue harvests specific to each tumour type across sites (this can be monitored using the PEACE sample tracker database)
- Number of tissue harvests performed on patients that are enrolled in other clinical and genomic studies (preference is for this type of tissue harvest)
- Compliance with Trial Specific Procedures (sample collection, processing and storage)
- The opening of new sites (depending on available funding)
- The closure of underperforming sites
- Arising publications
- Approval of proposals from within or outside the UK for access to collected samples with the intention of undertaking ethically approved and funded research. Proposals will be judged based on scientific merit and where possible collaborative initiative will be encouraged and supported.
- Supervise issues related to the negotiation and assignment of Intellectual Property (initially a set of principles and sharing of credit across contributing Centres could be agreed in consultation with the Lead Centre, and subsequently an Intellectual Property Governance group can be established)

10.5.3. Independent Data Monitoring Committee (IDMC)

The role of the IDMC is to provide independent advice on accrual and data returns. Meetings of the Committee will be held approximately every 12 months to review interim analyses or as necessary to address any issues.

Reviews will be undertaken to examine:

- Accrual rates
- Number of Tissue Harvests performed
- Data return
- Approved Research proposals

10.5.4. Role of UCL CTC

UCL CTC will be responsible for the day to day coordination and management of the study and will act as custodian of the data generated in the study (in behalf of UCL).

Docusign Envelope ID: AB97AAF1-C9D6-4C1D-9E5C-84B38883CE23

11. Withdrawal of Patients

In consenting to the study, patients or their nominated representatives/ person in a qualifying relationship are consenting to study assessments, follow-up, data collection and access to data already collected through other trial participation, blood and tissue sampling, access to previously excised tissue and tissue harvesting. If possible, we aim to replace patients who are withdrawn early (this can only be done during recruitment).

11.1. Discontinuation of Study Participation

A patient may be withdrawn from study participation whenever continued participation is no longer in the patient's best interests, but the reasons for doing so must be recorded. Reasons for discontinuing participation may include

- Patient or their representative/relation choice

In these cases patients remain within the study for the purposes of follow-up and data analysis.

If a patient or their representative expresses their wish to withdraw from study participation, sites should explain the importance of remaining on study follow-up, or failing this of allowing routine follow-up data to be used for study purposes and for allowing existing collected data and samples to be used. If the patient gives a reason for their withdrawal, this should be recorded.

11.2. Future Data Collection

If a patient or their representative explicitly states they do not wish to contribute further data or samples (either when alive or post humously) to the study this decision must be respected and recorded on the relevant eCRF. In this event details should be recorded in the patient's hospital records, no further eCRFs must be completed and no further data sent to UCL CTC.

11.3. Losses to Follow-Up

If a patient moves from the area, every effort should be made for the patient to be followed up at another participating study site and for this new site to take over the responsibility for the patient, or for follow-up via GP. Details of participating study sites can be obtained from the UCL CTC study team who must be informed of the transfer of care and follow up arrangements.

If a patient is lost to follow-up at a site every effort should be made to contact the patient's GP to obtain information on the patient's status.

Docusign Envelope ID: AB97AAF1-C9D6-4C1D-9E5C-84B38883CE23

12. STUDY CLOSURE

12.1. End of Study

End of study will be 30 September 2030.

Following this, UCL CTC will advise sites on the procedure for closing the study at the site.

Alternative arrangements will be considered nearer to the time to ensure continued access to the PEACE sample tracker database and sample collection (if samples still exist) as well as ensuring an appropriate Study Governance Board remains in existence to make decisions on sample provision for research projects.

12.2. Archiving of Study Documentation

At the end of the study, UCL CTC will archive securely all centrally held study related documentation for a minimum of 5 years. Arrangements for confidential destruction will then be made. It is the responsibility of PIs to ensure data and all essential documents relating to the study held at site are retained for a minimum of 5 years after the end of the study, in accordance with national legislation and for the maximum period of time permitted by the site.

Essential documents are those which enable both the conduct of the study and the quality of the data produced to be evaluated and show whether the site complied with the principles of GCP and all applicable regulatory requirements.

UCL CTC will notify sites when study documentation held at sites may be archived. All archived documents must continue to be available for inspection by appropriate authorities upon request.

12.3. Early Discontinuation of Study

The Study may be stopped before completion on the recommendation of the Study Governance Board or PEACE consortium.

Sites will be informed in writing by UCL CTC of reasons for early closure and the actions to be taken with regards the follow up of patients.

12.4. Withdrawal from Study Participation by a Site

Should a site choose to close to recruitment the PI must inform UCL CTC in writing. Follow up as per protocol must continue for all patients recruited into the study at that site and other responsibilities continue as per CTSA.

Docusign Envelope ID: AB97AAF1-C9D6-4C1D-9E5C-84B38883CE23

13. QUALITY ASSURANCE

The monitoring processes in section 10 above will form the mainstay of quality control within the study.

Established secure and confidential computerised systems for recording data are in place.

Investigators and study personnel will be made available for possible audits and inspections by the institutional review board or Ethics Committee.

All source documentation and the site study file will be available for inspection.

Docusign Envelope ID: AB97AAF1-C9D6-4C1D-9E5C-84B38883CE23

14. ETHICAL APPROVALS

In conducting the study, the Sponsor, UCL CTC and sites shall comply with the protocol and with all relevant guidance, laws and statutes, as amended applicable to the performance of clinical trials and research, including, but not limited to:

- UK Policy Framework for Heath and Social Care Research, issues by the Health Research Authority
- The principles of Good Clinical Practice
- Human Rights Act 1998
- Data Protection Act 2018
- General Data Protection Regulation (EU)2016/679 (GDPR)
- Freedom of Information Act 2000
- Human Tissue Act 2004
- Mental Capacity Act 2005
- the Research Governance Framework for Health and Social Care, issued by the UK Department of Health (Second Edition 2005) or the Scottish Health Department Research Governance Framework for Health and Community Care (Second Edition 2006)

14.1. Ethical Approval

The Study will be conducted in accordance with the World Medical Association Declaration of Helsinki entitled ‘Ethical Principles for Medical Research Involving Human Subjects’ (1996 version) and in accordance with the terms and conditions of the ethical approval given to the Study.

The Study has received a favourable opinion from the London – Dulwich Research Ethics Committee UCL CTC will submit Annual Progress Reports to the REC, which will commence one year from the date of ethical approval for the Study.

14.2. Specific Ethical Issues

Research on recently deceased patients has its own ethical considerations with the overriding need to balance the potential benefits from the research with moral obligations to the recently deceased. Cadavers should be treated in a manner that is consistent with respect for the value and dignity of the once-living person and researchers will need to be sensitive to cultural differences in views of proper treatment of the deceased. There are several points this study adheres to:

1. The research addresses a significant question and uses methods likely to produce valid results.
2. Coronial post-mortems will take precedence if space and manpower are limited.
3. Death will be independently determined by physicians not involved in the study.
4. Decisions pertaining to withdrawal of life support from an individual who may be eligible for the study will not be made by research physicians.

Docusign Envelope ID: AB97AAF1-C9D6-4C1D-9E5C-84B38883CE23

5. Procedures will be performed in a respectful manner.
6. Respect for the dead, their legacy and their living relatives and friends require that patient confidentiality is strictly adhered to.

14.3. Site Approvals

Evidence of institutional approvals/confirmation of capacity and capability for a study site must be provided to UCL CTC. Sites will only be activated when all necessary local approvals/confirmation of capacity and capacity for the study have been obtained.

14.4. Protocol Amendments

UCL CTC will be responsible for gaining ethical approval, for amendments made to the protocol and other study-related documents. Once approved, UCL CTC will ensure that all amended documents are distributed to sites and LCRNs as appropriate.

Site staff will be responsible for acknowledging receipt of documents and for implementing all amendments.

14.5. Patient Confidentiality & Data Protection

Patient identifiable data, including, date of birth, ethnicity and NHS number will be required for the registration process and will be provided to UCL CTC. UCL CTC will preserve patient confidentiality and will not disclose or reproduce any information by which patients could be identified. Data will be stored in a secure manner and UCL CTC studies are registered in accordance with the Data Protection Act 2018 and GDPR with the Data Protection Officer at UCL.

15. SPONSORSHIP AND INDEMNITY

15.1. Sponsor Details

Sponsor Name:	University College London
Address:	Joint Research Office<br>4th Floor, West<br>250 Euston Road<br>London<br>NW1 2PG
Contact:	Managing Director, UCLH/UCL Research
Tel:	020 3447 9995/2178 (unit admin)

15.2. Indemnity

University College London holds insurance against claims from participants for injury caused by their participation in the clinical trial. Participants may be able to claim compensation if they can prove that UCL has been negligent. However, if this clinical trial is being carried out in a hospital, the hospital continues to have a duty of care to the participant of the clinical trial. University College London does not accept liability for any breach in the hospital's duty of care, or any negligence on the part of hospital employees. This applies whether the hospital is an NHS Trust or otherwise.

Hospitals selected to participate in this clinical trial shall provide clinical negligence insurance cover for harm caused by their employees and a copy of the relevant insurance policy or summary shall be provided to University College London, upon request.

Docusign Envelope ID: AB97AAF1-C9D6-4C1D-9E5C-84B38883CE23

16. FUNDING

The PEACE study is funded by a Cancer Research UK Centre Accelerator Award.

As PEACE is incorporated into specific research studies, PEACE will be funded by various dedicated funders of those research studies.

Docusign Envelope ID: AB97AAF1-C9D6-4C1D-9E5C-84B38883CE23

17. PUBLICATION POLICY

Proposals for publication should be sent to Study Governance Board for review at least 30 days prior to submission. Upon request any party will delay publication for up to 90 days to allow for publications to be reviewed.

Professor Charles Swanton and Professor Mariam Jamal-Hanjani should be named co-senior authors on papers derived from PEACE. UCL CTC should be named co-authors on papers specifically focused on the PEACE study. All papers derived from PEACE should name the PEACE consortium in the list of co-authors, for example, ‘PEACE consortium, Charles Swanton and Mariam Jamal-Hanjani’. Where possible, journals should index all members of the consortium against publications on PubMed. If this is not possible, members can be named in an appendix or supplementary file. UCL CTC will provide an up to date list of members for publication.

Docusign Envelope ID: AB97AAF1-C9D6-4C1D-9E5C-84B38883CE23

18. REFERENCES

1. Rubin, M. A. *et al.* Rapid ('warm') autopsy study for procurement of metastatic prostate cancer. *Clinical Cancer Research* **6,** 1038–1045 (2000).
2. Embuscado, E. E. *et al.* Immortalizing the complexity of cancer metastasis: genetic features of lethal metastatic pancreatic cancer obtained from rapid autopsy. *Cancer Biol Ther* **4,** 548–554 (2005).
3. Juric, D. *et al.* Convergent loss of PTEN leads to clinical resistance to a PI(3)Kα inhibitor. *Nature* **518,** 240–244 (2015).
4. Cummings, M. C. *et al.* Metastatic progression of breast cancer: insights from 50 years of autopsies. *J. Pathol* **232,** 23–31 (2013).
5. Liu, W. *et al.* Copy number analysis indicates monoclonal origin of lethal metastatic prostate cancer. *Nat Med* **15,** 559–565 (2009).
6. Borthwick, N. J. *et al.* The biology of micrometastases from uveal melanoma. *Journal of Clinical Pathology* **64,** 666–671 (2011).
7. Jamal-Hanjani, M. *et al.* Tracking genomic cancer evolution for precision medicine: the lung TRACERx study. *PLoS Biol.* **12,** e1001906 (2014).
8. Gerlinger, M. *et al.* Intratumor heterogeneity and branched evolution revealed by multiregion sequencing. *N Engl J Med* **366,** 883–892 (2012).
9. Paik, P. K. *et al.* Next-Generation Sequencing of Stage IV Squamous Cell Lung Cancers Reveals an Association of PI3K Aberrations and Evidence of Clonal Heterogeneity in Patients with Brain Metastases. *Cancer Discovery* **5,** 610–621 (2015).
10. Shah, S. P. *et al.* Mutational evolution in a lobular breast tumour profiled at single nucleotide resolution. *Nature* **461,** 809–813 (2009).
11. Campbell, P. J. *et al.* The patterns and dynamics of genomic instability in metastatic pancreatic cancer. *Nature* **467,** 1109–1113 (2010).
12. Yachida, S. *et al.* Distant metastasis occurs late during the genetic evolution of pancreatic cancer. *Nature* **467,** 1114–1117 (2010).
13. Kogita, A. *et al.* Inter- and intra-tumor profiling of multi-regional colon cancer and metastasis. *Biochem. Biophys. Res. Commun.* **458,** 52–56 (2015).
14. Tan, I. B. *et al.* High-depth sequencing of over 750 genes supports linear progression of primary tumors and metastases in most patients with liver-limited metastatic colorectal cancer. *Genome Biology* **16,** 32 (2015).
15. Nadauld, L. D. *et al.* Metastatic tumor evolution and organoid modeling implicate TGFBR2 as a cancer driver in diffuse gastric cancer. *Genome Biology* **15,** 428 (2014).
16. Haffner, M. C. *et al.* Tracking the clonal origin of lethal prostate cancer. *J. Clin. Invest.* **123,** 4918–4922 (2013).
17. Hong, M. K. H. *et al.* Tracking the origins and drivers of subclonal metastatic expansion in prostate cancer. *Nature Communications* **6,** 6605 (2015).
18. Gundem, G. *et al.* The evolutionary history of lethal metastatic prostate cancer. *Nature* **520,** 353–357 (2015).
19. Wu, X. *et al.* Clonal selection drives genetic divergence of metastatic medulloblastoma. *Nature* **482,** 529–533 (2012).
20. Snuderl, M. *et al.* Mosaic Amplification of Multiple Receptor Tyrosine Kinase Genes in Glioblastoma. *Cancer Cell* **20,** 810–817 (2011).

Docusign Envelope ID: AB97AAF1-C9D6-4C1D-9E5C-84B38883CE23

21. Sottoriva, A. *et al.* Intratumor heterogeneity in human glioblastoma reflects cancer evolutionary dynamics. *Proc. Natl. Acad. Sci. U.S.A.* **110,** 4009–4014 (2013).
22. Suzuki, H. *et al.* Mutational landscape and clonal architecture in grade II and III gliomas. *Nature Genetics* **47,** 458–468 (2015).
23. Kumar, A. *et al.* Deep sequencing of multiple regions of glial tumors reveals spatial heterogeneity for mutations in clinically relevant genes. *Genome Biology* **15,** 530 (2014).
24. McGranahan, N. *et al.* Clonal status of actionable driver events and the timing of mutational processes in cancer evolution. *Science Translational Medicine* **7,** 283ra54–283ra54 (2015).
25. de Bruin, E. C. *et al.* Spatial and temporal diversity in genomic instability processes defines lung cancer evolution. *Science* **346,** 251–256 (2014).
26. Kanu, N. *et al.* SETD2 loss-of-function promotes renal cancer branched evolution through replication stress and impaired DNA repair. *Oncogene* **34,** 5699–5708 (2015).
27. Murtaza, M. *et al.* Non-invasive analysis of acquired resistance to cancer therapy by sequencing of plasma DNA. *Nature* **497,** 108–112 (2014).
28. Diehl, F. *et al.* Circulating mutant DNA to assess tumor dynamics. *Nat Med* **14,** 985–990 (2008).
29. Diehl, F. *et al.* Detection and quantification of mutations in the plasma of patients with colorectal tumors. *Proceedings of the National Academy of Sciences* **102,** 16368–16373 (2005).
30. Dawson, S.-J. *et al.* Analysis of Circulating Tumor DNA to Monitor Metastatic Breast Cancer. *N Engl J Med* **368,** 1199–1209 (2013).
31. Diaz, L. A. *et al.* The molecular evolution of acquired resistance to targeted EGFR blockade in colorectal cancers. *Nature* **486,** 537–540 (2012).
32. Carreira, S. *et al.* Tumor clone dynamics in lethal prostate cancer. *Science Translational Medicine* **6,** 254ra125–254ra125 (2014).
33. Siravegna, G. *et al.* Clonal evolution and resistance to EGFR blockade in the blood of colorectal cancer patients. *Nat Med* **21,** 795–801 (2015).
34. Garcia-Murillas, I. *et al.* Mutation tracking in circulating tumor DNA predicts relapse in early breast cancer. *Science Translational Medicine* **7,** 302ra133–302ra133 (2015).
35. Murtaza, M. *et al.* Multifocal clonal evolution characterized using circulating tumour DNA in a case of metastatic breast cancer. *Nature Communications* **6,** 8760 (2015).
36. Bettegowda, C. *et al.* Detection of Circulating Tumor DNA in Early- and Late-Stage Human Malignancies. *Science Translational Medicine* **6,** 224ra24–224ra24 (2014).
37. Antonarakis, E. S. *et al.* AR-V7 and Resistance to Enzalutamide and Abiraterone in Prostate Cancer. *N Engl J Med* 140903140143003 (2014). doi:10.1056/NEJMoa1315815
38. Ni, X. *et al.* Reproducible copy number variation patterns among single circulating tumor cells of lung cancer patients. *Proc. Natl. Acad. Sci. U.S.A.* **110,** 21083–21088 (2013).
39. Heitzer, E. *et al.* Complex tumor genomes inferred from single circulating tumor cells by array-CGH and next-generation sequencing. *Cancer Research* **73,** 2965–2975 (2013).
40. Hodgkinson, C. L. *et al.* Tumorigenicity and genetic profiling of circulating tumor cells in small-cell lung cancer. *Nat Med* (2014). doi:10.1038/nm.3600

Docusign Envelope ID: AB97AAF1-C9D6-4C1D-9E5C-84B38883CE23

41. Lohr, J. G. *et al.* Whole-exome sequencing of circulating tumor cells provides a window into metastatic prostate cancer. *Nature Biotechnology* 1–8 (2014). doi:10.1038/nbt.2892
42. Dunn, G. P., Bruce, A. T., Ikeda, H., Old, L. J. & Schreiber, R. D. Cancer immunoediting: from immunosurveillance to tumor escape. *Nat. Immunol.* **3,** 991–998 (2002).
43. Matsushita, H. *et al.* Cancer exome analysis reveals a T-cell-dependent mechanism of cancer immunoediting. *Nature* **482,** 400–404 (2012).
44. DuPage, M., Mazumdar, C., Schmidt, L. M., Cheung, A. F. & Jacks, T. Expression of tumour-specific antigens underlies cancer immunoediting. *Nature* **482,** 405–409 (2012).
45. van den Boorn, J. G. & Hartmann, G. Turning Tumors into Vaccines: Co-opting the Innate Immune System. *Immunity* **39,** 27–37 (2013).
46. Fisher, R., Pusztai, L. & Swanton, C. Cancer heterogeneity: implications for targeted therapeutics. *British Journal of Cancer* **108,** 479–485 (2013).
47. Peggs, K. S., Segal, N. H. & Allison, J. P. Targeting Immunosupportive Cancer Therapies: Accentuate the Positive, Eliminate the Negative. *Cancer Cell* **12,** 192–199 (2007).
48. Segal, N. H. *et al.* Epitope landscape in breast and colorectal cancer. *Cancer Research* **68,** 889–892 (2008).
49. Jamal-Hanjani, M., Thanopoulou, E., Peggs, K. S., Quezada, S. A. & Swanton, C. Tumour heterogeneity and immune-modulation. *Current Opinion in Pharmacology* **13,** 497–503 (2013).
50. Gooden, M. J. M., de Bock, G. H., Leffers, N., Daemen, T. & Nijman, H. W. The prognostic influence of tumour-infiltrating lymphocytes in cancer: a systematic review with meta-analysis. *British Journal of Cancer* **105,** 93–103 (2011).
51. Loi, S. *et al.* Prognostic and predictive value of tumor-infiltrating lymphocytes in a phase III randomized adjuvant breast cancer trial in node-positive breast cancer comparing the addition of docetaxel to doxorubicin with doxorubicin-based chemotherapy: BIG 02-98. *J. Clin. Oncol.* **31,** 860–867 (2013).
52. Sato, E. *et al.* Intraepithelial CD8+ tumor-infiltrating lymphocytes and a high CD8+/regulatory T cell ratio are associated with favorable prognosis in ovarian cancer. *Proceedings of the National Academy of Sciences* **102,** 18538–18543 (2005).
53. Nelson, B. H. The impact of T-cell immunity on ovarian cancer outcomes. *Immunol. Rev.* **222,** 101–116 (2008).
54. Hwang, W.-T., Adams, S. F., Tahirovic, E., Hagemann, I. S. & Coukos, G. Prognostic significance of tumor-infiltrating T cells in ovarian cancer: a meta-analysis. *Gynecol. Oncol.* **124,** 192–198 (2012).
55. Yamada, N. *et al.* CD8+ tumor-infiltrating lymphocytes predict favorable prognosis in malignant pleural mesothelioma after resection. *Cancer Immunol. Immunother.* **59,** 1543–1549 (2010).
56. Yewdell, J. W. & Bennink, J. R. Immunodominance in major histocompatibility complex class I-restricted T lymphocyte responses. *Annu. Rev. Immunol.* **17,** 51–88 (1999).
57. Heemskerk, B., Kvistborg, P. & Schumacher, T. N. M. The cancer antigenome. *The EMBO Journal* **32,** 194–203 (2012).

Docusign Envelope ID: AB97AAF1-C9D6-4C1D-9E5C-84B38883CE23

APPENDIX 1: ABBREVIATIONS

**cfDNA**	Circulating free Tumour DNA
**CI**	Chief Investigator
**CR**	Complete response
**CRF**	Case Report Form
**CT**	Computerised Tomography
**CTCs**	Circulating Tumour Cells
**CTSA**	Clinical Study Site Agreement
**CUP**	Cancer of unknown primary
**CXR**	Chest X-Ray
**DFS**	Disease Free Survival
**DNA**	Deoxyribonucleic Acid
**DPA**	Data Protection Act
**ECG**	Electrocardiogram
**eCRF**	Electronic Case Report Form
**FFPE**	Formalin fixed, paraffin embedded
**GBM**	Glioblastoma multiforme
**GDPR**	General Data Protection Regulation
**GI**	Gastrointestinal
**GSTT**	Guy’s and St. Thomas’ Hospital
**GU**	Genitourinary
**H&E**	Hematoxylin and Eosin stain
**HTA**	Human Tissue Authority
**ICH GCP**	International Conference of Harmonisation-Good Clinical Practice
**ICPV**	Independent Cancer Patients’ Voice
**ICR**	Institute of Cancer Research
**IDMC**	Independent Data Monitoring Committee
**LCRN**	Local Clinical Research Network
**MHC**	Major histocompatibility complex
**MRI**	Magnetic Resonance Image
**NCRI**	National Cancer Research Institute
**NCRN**	National Cancer Research Network
**OC**	OpenClinica
**OS**	Overall Survival
**PD**	Progressive Disease
**PFS**	Progression Free Survival
**PI**	Principal Investigator
**PIK**	Phosphatidylinositol-4,5-Bisphosphate 3-Kinase
**PTEN**	Phosphatase and tensin homolog
**REC**	Research Ethics Committee
**RECIST**	Response Evaluation Criteria in Solid Tumours
**RMH**	Royal Marsden Hospital
**SGB**	Study Governance Board
**SNV**	Single Nucleotide Variant
**SSA**	Site Specific Assessment
**TIL**	Tumour infiltrating lymphocytes

Docusign Envelope ID: AB97AAF1-C9D6-4C1D-9E5C-84B38883CE23

**TMA**	Tissue microarray
**TMF**	Trial (study) Master File
**UCL**	University College London
**UCL CI**	UCL Cancer Institute
**UCL CTC**	CR UK and UCL Cancer Trials Centre
**UCLH**	University College London Hospital

Docusign Envelope ID: AB97AAF1-C9D6-4C1D-9E5C-84B38883CE23

APPENDIX 2: PROTOCOL VERSION HISTORY

**Protocol:**			
**Version no.**	**Date**	**Amendment no.**	**Summary of main changes from previous version**
1.0	24/05/2013	N/A	N/A
2.0	18/12/2013	1.0	• To include additional blood samples to be taken at an existing time point (i.e CTC and cfDNA blood to be taken in addition to the existing germline blood sample)
3.0	10/03/2014	2.0	• Amended from single to multi-centre study and from a pilot study to a prospective observational study<br>• Outside hospital deaths - allow transfer of bodies from other sites/outside of a hospital to a participating study site for the post mortem<br>• Increase in total blood collection from 40 to 50 ml<br>• Amended wording to state eyes donations can be made to local eye banks should they exist (previously donation was specifically to (Moorfield's Eye bank)
4.0	11/11/2016	4.0	• PEACE acronym changed to Posthumous Evaluation of Advanced Cancer Environment.<br>• Sponsor changed from UCLH to UCL (as now a multi-centre study)<br>• Contact details updated for UCL CTC and the PEACE consortium<br>• Generic study pathways added<br>• Target accrual changed from 50 to 500<br>• Eligibility updated: Confirmed diagnosis of any form of solid malignancy with metastatic disease (where the site of origin is known or unknown), with the exception of primary brain tumour in which there may not be evidence of metastatic disease. New exclusion added: history of intravenous drug abuse within the last 5 years<br>• Background updated in keeping with new literature.<br>• Potential future research projects added to include in vivo models and cell line derivation models<br>• Consent process may be provided by either patient (as in previous protocol) or in addition, 'Nominated representative' or 'Person in qualifying relationship' with the patient.<br>• Consent process updated – patients should be given adequate time to consider the trial – a minimum of 24 hours is no longer specified. Also a copy of the signed consent form will be submitted to UCL CTC.<br>• Blood sampling updated – samples will be collected at baseline and at up to 4 time points. Sampling may occur at the time of tissue sampling if samples were not collected prior to death. Plasma analyses updated to include cytokine and metabolite analyses. Additional blood sampling for immunology and T-cell and B-cell receptor sequencing.

Docusign Envelope ID: AB97AAF1-C9D6-4C1D-9E5C-84B38883CE23

4.0		4.0	• Wording added to ensure a copy of the consent form and other documentation as required by local policy is made available to mortuary technician and pathologist performing the post-mortem tissue sampling prior to moving the body and any subsequent tissue collection. Wording also added to ensure the consent form is checked to verify if the patient wished to limit the sampling of tissue post-mortem<br>• Wording added to specify tissue sample should ideally occur within 24 hours after death<br>• Where available, sample collection at post-mortem to include collection of bone marrow samples and whole organs, such as the bladder and prostate, may be removed.<br>• If present at the time of tissue collection after death, the following samples may also be collected: cerebrospinal fluid, pleural fluid, pericardial fluid, peritoneal fluid and urine.<br>• Where site facilities permit, imaging will be performed prior to the tissue sampling procedure at post-mortem. Previous scans will also be requested including the imaging that was used to guide the tissue sampling at the post-mortem (if applicable). Copies of any images used to guide tissue sampling will be collected centrally at UCL CTC.<br>• Details added regarding the use of barcodes and a sample tracking database and an electronic database for data capture (eCRFs)<br>• Details added regarding creation of a digital archive<br>• End of study definition added: 5 years following activation of the grant or upon reaching 500 post-mortems (whichever is soonest)<br>• Additional information added regarding oversight committees.<br>• Indemnity wording updated<br>• Publication policy wording updated<br>• Glossary added
4.1	09/12/2016	4.0	• Wording updated to say that the prostate is the only organ that may be removed in its entirety<br>• Wording updated to clarify that a nominated rep or person in a qualifying relationship can only consent after the patient's death<br>• Wording added re witness signature for nominated rep or person in a qualifying relationship consent form
4.2	09/01/2017	4.1	• Admin changes, wording updated to re-include details in blood sample collection section and amendment numbers updated in protocol version history table

Docusign Envelope ID: AB97AAF1-C9D6-4C1D-9E5C-84B38883CE23

4.3	08/01/2017	5.0	• Change in trial coordinator details<br>• Removal of consortium list. UCL CTC to be contacted for up to date list<br>• Clarification in exclusion criteria; patients only excluded if they have diagnosed high risk infection<br>• Clarification that sampling will be guided by imaging (pre and post death), findings at tissue harvest and high clinical suspicion<br>• Addition of central and collaborating laboratories<br>• Amended wording from 'post-mortem' to 'tissue harvest in post-mortem setting' throughout<br>• Addition of permission to remove brain in its entirety during tissue sampling<br>• Addition of home visits to collect consent, follow up patients and collect blood samples<br>• Confirmation that PEACE will not be contributing towards any funeral costs<br>• Addition that optional consent will be requested for the tissue sampling procedure to be filmed<br>• Analysis from PEACE may be combined with analysis performed on data from other trials/studies<br>• Additional potential analyses; Blood platelets mRNA, IncrRNA and microRNA expression analyses by NGS or RT-PCR methods, DNA analysis in blood platelets by NHS or RT-PCR methods, analysis of normal tissue where appropriate.
5.0	20/01/2020	6.0	• N/A not approved by ethics committee
6.0	24/04/2020	8.0	• N/A not approved by ethics committee
7.0	14/05/2020	9.0	• Minor admin changes throughout<br>• Change of study CI<br>• Change of trial coordinator details<br>• Exclusion criteria updated to allow patients with high risk infections to be included in the study if the patient is of particular scientific interest and is agreed in advance with local mortuary staff and pathologist<br>• Collaborating translational research laboratories updated<br>• New planned sample analysis added to section 8.4<br>• Requirement to send UCL CTC consent forms removed<br>• Immunology blood sample increased from 20ml to 40ml<br>• Removal of follow up timepoint limit<br>• Clarification that consent can be obtained from person in qualifying relationship or nominated representative at home after the patient's death, in line with trust policies<br>• Clarification that witness signature not required if not mandatory for the mortuary<br>• Clarification on mortuary staff viewing consent form<br>• Removal of blood sampling limit to allow blood samples to be obtained both before and after death<br>• Recruitment selection criteria updated<br>• Clarification of sample storage<br>• Change of location of H&E slide digital hub scanning<br>• Addition of scan reports to be collected alongside scans<br>• Clarification of on-site monitoring<br>• Additional documents added for submission under central monitoring<br>• Data protection and GDPR updates<br>• Information on IDMC added<br>• Wording added to publication policy<br>• Appendix 2 added which includes COVID-19 sub-study

Docusign Envelope ID: AB97AAF1-C9D6-4C1D-9E5C-84B38883CE23

7.1	07/08/2020	9.1	• Typo corrected in Section 8.1 - Total blood amount incorrectly stated as 73, changed to 93ml<br>• Clarified that 40ml immunological analysis bloods are only for patients who fit following criteria: - Patient is also participating in the Lung TRACERx study, - Selected lung patients recruited at UCLH/UCLH affiliated sites<br>• Typo corrected in Section 8.1 – from “at up to four time points” to “at subsequent follow-up visits”
7.2	07/12/2020	9.2	• Section 8.1 - Clarified that 40ml immunological analysis bloods are only for patients who fit following criteria: “only required for patients who are also participating in the Lung TRACERx study, and selected lung patients”. The specification that these lung patients must be from UCLH or UCLH-affiliate sites has been removed.<br>• Section 10.2 – added reference to TSP 6 – Photograph Capture, Labelling and Transfer
7.3	23/11/2021	9.4	• Removal of Director/Deputy Director signature as no longer required<br>• Fax numbers removed throughout<br>• Definition of end of study updated<br>• Collaborating translational research laboratories section added to<br>• Added option of videoconference where applicable<br>• remote consent process added<br>• remote follow up assessment added<br>• removal of reference to mortuary not keeping consent form<br>• wording updated relating to UK and EU clinical trial regulations<br>• Sponsor contact role updated<br>• Publication policy updated<br>• Other minor updates/corrections throughout
8.0	23/11/2023	10.0	• CRUK and UCL Cancer Trials Centre signatory update<br>• Sponsor contact role updated<br>• Definition of end of study updated<br>• Germline blood collected at follow up if not collected at baseline<br>• Wording updated relating to where tissue sampling may be performed<br>• Wording updated relating to sample storage<br>• Addition of IPS cells analysis to section 8.4<br>• Demographic data collection added<br>• Sponsor address updated<br>Other minor updates/corrections throughout

Docusign Envelope ID: AB97AAF1-C9D6-4C1D-9E5C-84B38883CE23

9.0	12/08/2024	11.0	• PEACE CI and CRUK and UCL Cancer Trials Centre signatory update<br>• Summary of study design updated to reflect updates made throughout<br>• Patient pathways updated inline with updates hospital/study processes<br>• Removal of reference to specific cancer programmes.<br>• Reference to PEACE supporting TRACERx EVO study throughout<br>• Removal of Evolution of Glioblastoma background information<br>• Removal of sites 10-15 tissue harvest target<br>• Clarification of wording re tumour organoid models<br>• Removal of reference to tissue harvests being filmed<br>• Removal of reference re Scotland specific consent<br>• Selection of patients updated<br>• Recruitment selection updated<br>• OpenClinica link added<br>• CTC blood sample requirement updated<br>• Reference to patients being scanned after death removed<br>• Wording relating to scan and scan report transfer updated<br>• Wording relating to place of tissue harvest clarified<br>• SGB definition and role updated<br>• SOG definition and role added<br>• End of study date updated<br>• Funding updated<br>• Appendix 2- PEACE COVID-19 Sub-Study removed
9.1	11/09/2024	11.1	• Anticipated duration of recruitment clarified
